# ﻿New and interesting species of *Penicillium* (Eurotiomycetes, Aspergillaceae) in freshwater sediments from Spain

**DOI:** 10.3897/mycokeys.86.73861

**Published:** 2022-02-01

**Authors:** Daniel Torres-Garcia, Josepa Gené, Dania García

**Affiliations:** 1 Universitat Rovira i Virgili, Unitat de Micologia, Facultat de Medicina i Ciències de la Salut and IISPV, 43201-Reus, Spain Universitat Rovira i Virgili Reus Spain

**Keywords:** 5 new species, Ascomycota, Eurotiales, fluvial sediments, phylogeny, species delimitation, taxonomy

## Abstract

*Penicillium* species are common fungi found worldwide from diverse substrates, including soil, plant debris, food products and air. Their diversity in aquatic environments is still underexplored. With the aim to explore the fungal diversity in Spanish freshwater sediments, numerous *Penicillium* strains were isolated using various culture-dependent techniques. A preliminary sequence analysis of the β-tubulin (*tub*2) gene marker allowed us to identify several interesting species of *Penicillium*, which were later characterized phylogenetically with the barcodes recommended for species delimitation in the genus. Based on the multi-locus phylogeny of the internal transcribed spacer region (ITS) of the ribosomal DNA, and partial fragments of *tub*2, calmodulin (*cmdA*), and the RNA polymerase II largest subunit (*rpb*2) genes, in combination with phenotypic analyses, five novel species are described. These are *P.ausonanum* in sectionLanata-Divaricata, *P.guarroi* in sect.Gracilenta, *P.irregulare* in sect.Canescentia, *P.sicoris* in sect.Paradoxa and *P.submersum* in sect.Robsamsonia. The study of several isolates from samples collected in different locations resulted in the reinstatement of *P.vaccaeorum* into sectionCitrina. Finally, *P.heteromorphum* (sect.Exilicaulis) and *P.tardochrysogenum* (sect.Chrysogena) are reported, previously only known from Antarctica and China, respectively.

## ﻿Introduction

Fungi make up a significant component of the benthic microbial biomass in freshwater ecosystems, and play a pivotal role in decomposing organic matter in river habitats (Pascoal and Cassio 2004; Zhang et al. 2013). In general, representatives of Ascomycota account for the largest number of species recovered from water systems (Kelley et al. 2001; Liu et al. 2015; Khomich et al. 2017; Sutcliffe et al. 2018; Samson et al. 2020), their asexual forms with stauro- or scolecoconidia (aquatic hyphomycetes) being the most representative fungal group in freshwater environments (Pascoal et al. 2005; Jones and Pang 2012). Although common Ascomycota like *Penicillium* species are not considered aquatic fungi, they are one of the most common genera identified from freshwater (Pitt 1979; Hageskal et al. 2006; Sonjak et al. 2006; Samson et al. 2010; Heo et al. 2019; Pangging et al. 2019) and recently were also reported in marine environments (Kirichuk et al. 2017; Gonçalves et al. 2019; Grossart et al. 2019). However, in almost all of those reports, little attention has been paid to the study of river sediments as a reservoir of taxonomically interesting penicillia.

*Penicillium* (Eurotiomycetes, Eurotiales, Aspergillaceae) is a ubiquitous genus recovered from a wide range of substrates–including soil, plant material, indoor and outdoor air environments, a variety of food products, herbivore dung and water (Visagie et al. 2014a; Barbosa et al. 2018; Grossart et al. 2019; Guevara-Suárez et al. 2020; Rodríguez-Andrade et al. 2021). *Penicillium* species have a great impact on human life as agents of food spoilage, causal agents of pre- and postharvest diseases on crops (Frisvad and Samson 2004; Pitt and Hocking 2009; Samson et al. 2010), and their ability to produce toxic compounds like mycotoxins (Frisvad and Samson 2004; Perrone and Susca 2017), which can be a serious threat to human and animal health worldwide. On the contrary, positive impacts of these fungi include their use in food fermentations, the production of many bioactive compounds of medical interest (antimicrobial agents, immunosuppressants), and the production of enzymes with a variety of industrial applications (Houbraken et al. 2014; Visagie et al. 2014a; Abdelwahab et al. 2018; Park et al. 2019; Ogaki et al. 2020a, b). Other recent applications are the potential use of some species as biocontrol agents against plant pathogens or for bioremediation in polluted environments (Guijarro et al. 2017; Cecchi et al. 2019; Behera et al. 2020; Thambugala et al. 2020; Liang et al. 2021). The discovery of novel lineages in the genus *Penicillium*, therefore, represents an opportunity to find and characterize new fungi or molecules with a wide range of applications. As an example, more than 390 novel natural products have been isolated in recent years from marine-derived *Penicillium* fungi, many of which possess biological activity with potential application in new drug developments (Yang et al. 2021).

Limitations in establishing species boundaries by morphological and even molecular data are well-known in Ascomycota, mainly in those genera that comprise a huge number of species such as *Aspergillus*, *Fusarium* and *Trichoderma*, among many others. Therefore, the use of an integrative taxonomy that combines morphometric, phylogenetic, chemical and ecological data provides support for accurate species delimitation and formal description of novel taxa (Haelewaters et al. 2018; Aime et al. 2021; Maharachchikumbura et al 2021). Species delimitation in *Penicillium* is currently based on an integrative or polyphasic approach, which usually includes morphological features, extrolite profiles (when available), and multi-locus phylogenies (Visagie et al. 2014a). Phylogenetic markers used for this purpose include β-tubulin (*tub*2), the internal transcribed spacer region (ITS) of the ribosomal RNA gene (rDNA) and the calmodulin (*cmdA*) and the RNA polymerase II largest subunit (*rpb*2) genes. More than 480 species are accepted in the genus, distributed across 32 sections (Houbraken and Samson 2011; Visagie et al. 2014a; Houbraken et al. 2020). More recently, in a phylogenetic re-evaluation of various genera in the order Eurotiales, Houbraken et al. (2020) reinstated a series classification, and in particular for *Penicillium* recognized 89 series, contributing in this way to work easily with phylogenetic clades instead of large sections.

In this context, in an ongoing study of microfungal diversity from freshwater sediments collected from rivers or streams in different Spanish regions, we found several isolates of *Penicillium*, which could represent putative new or uncommon species of penicillia based on the sequence analysis of the recommended secondary identification marker *tub*2 (Visaige et al. 2014a). The present work aims to resolve the taxonomy of those isolates by using the above-mentioned polyphasic approach and following the Genealogical Concordance Phylogenetic Species Recognition (GCPSR) criterion (Taylor et al. 2000).

## ﻿Materials and methods

### ﻿Sampling and fungal isolation

Sediment samples were collected between February 2018 and December 2020 from rivers and streams in natural and rural areas from various Spanish provinces (Baleares, Barcelona, Lleida, Madrid, and Tarragona) (Table 1). Sterile 100 ml plastic containers were used for collecting samples from the bunk beds or edges of the rivers selected ca 10 cm below the surface layer. Samples were transported in a refrigerated container and immediately processed in the laboratory. Samples were shaken vigorously in the same containers and, after leaving them for 1 min to settle, the water was decanted, and the sediment poured onto several layers of sterile filter paper on plastic trays to remove any water excess (Ulfig et al. 1997). To isolate a wide range of fungal species, each sample was cultured as follows: 1 g of sediment was distributed across three Petri dishes and mixed with dichloran rose-bengal-chloramphenicol agar (DRBC; 2.5 g peptone, 5 g glucose, 0.5 g KH2PO4, 0.25 g MgSO4, 12.5 mg Rose Bengal, 100 mg chloramphenicol, 1 mg dichloran, 10 g agar, 500 mL distilled water) melted at 45 °C; and in parallel, another 1 g was distributed in three other Petri dishes and mixed with melted potato dextrose agar (PDA; Pronadisa) supplemented with 2 g/L of chloramphenicol and 2 g/L of cycloheximide. Once solidified, the cultures were incubated at room temperature (22–25 °C) and examined weekly with a stereomicroscope for up to 4–5 weeks. To achieve pure cultures, conidia of *Penicillium* colonies were transferred with a sterile dissection needle to plates with PDA supplemented with chloramphenicol and incubated at 25 °C in darkness.

**Table 1.** Strain information and GenBank/EMBL accession numbers of the *Penicillium* species investigated in this study.

Species	Section	Strain no.^1^	Substrate and Origin	GenBank nucleotide accession no.^2^:				Citation
				*tub*2	*cmdA*	ITS	*rpb*2	
***P.ausonanum***	***Lanata–Divaricata***	**FMR 16948^T^**	**Fluvial sediment, stream of the Guilleries National Park, Barcelona, Catalonia, Spain**	**LR655809**	**LR655810**	**LR655808**	**LR655811**	**This study**
***P.guarroi***	***Gracilenta***	**FMR 17747^T^**	**Fluvial sediment, Brugent River, Tarragona, Catalonia, Spain**	**LR814134**	**LR814140**	**LR814139**	**LR814145**	**This study**
*P.heteromorphum*	*Exilicaulis*	CBS 226.89^T^	Soil, China	KJ834455	KP016786	KC411702	JN406605	Visagie et al. 2014a
	*Exilicaulis*	**FMR 18043**	**Fluvial sediment, stream of the Cadí–Moixerò Natural Park, Lleida, Catalonia, Spain**	**LR861780**	**LR861782**	**LR861783**	**LR861784**	**This study**
***P.irregulare***	***Canescentia***	**FMR 17859^T^**	**Fluvial sediment, Miraflores River, Community of Madrid, Spain**	**LR814144**	**LR814151**	**LR814181**	**LR814182**	**This study**
*P.sanguifluum*	*Citrina*	CBS 127032^T^	Soil, Calahonda, Costa del Sol, Spain	JN606819	JN606555	JN617681	–	Houbraken et al. 2011
	*Citrina*	**FMR 17617**	**Fluvial sediment, Mallorca Island, Spain**	**LR861778**	**LR861781**	**LR861779**	–	**This study**
	*Citrina*	**FMR 17619**	**Fluvial sediment, Mallorca Island, Spain**	**OU375375**	–	–	–	**This study**
***P.sicoris***	***Paradoxa***	**FMR 18076^T^**	**Fluvial sediment, Segre River, Lleida, Catalonia, Spain**	**LR884494**	**LR884496**	**LR884497**	**LR884495**	**This study**
***P.submersum***	***Robsamsonia***	**FMR 17140^T^**	**Fluvial sediment, stream of the Montsant Natural Park, Tarragona, Catalonia, Spain**	**LR814187**	**LR814188**	**LR814194**	**LR814195**	**This study**
*P.tardochrysogenum*	*Chrysogena*	CBS 132200**^T^**	Soil, McMurdo Dry Valley, Antarctica	JX996898	JX996239	JX997028	JX996634	Houbraken et al. 2012
	*Chrysogena*	**FMR 17137**	**Fluvial sediment, stream of the Montsant Natural Park, Tarragona, Catalonia, Spain**	**HG996463**	**HG996465**	**HG996464**	–	**This study**
*P.vaccaeorum*	*Citrina*	CBS 148.83^T^	Sandy soil under pine tree, Valladolid, Spain	JN606846	JN606543	MH861558	–	Houbraken et al. 2011
	*Citrina*	**FMR 17967**	**Fluvial sediment, Basque Country, Spain**	**LR814226**	**LR814227**	**LR814235**	–	**This study**
	*Citrina*	**FMR 17531**	**Fluvial sediment, stream of Montseny National Park, Barcelona, Spain**	**LR814203**	**LR814204**	**LR814213**	–	**This study**
	*Citrina*	**FMR 17534**	**Fluvial sediment, stream of Montseny National Park, Barcelona, Spain**	**OU375168**	**OU375273**	**OU375272**	–	**This study**
	*Citrina*	**FMR 17616**	**Fluvial sediment, stream of Serra de Tramontana, Mallorca, Spain**	**LR814212**	**LR814218**	**LR814217**	–	**This study**
	*Citrina*	**FMR 18100**	**Fluvial sediment, Segre River, Lleida, Spain**	**LR814234**	**LR814242**	**LR814241**	–	**This study**
	*Citrina*	**FMR 18123**	**Fluvial sediment, Segre River, Lleida, Spain**	**LR814265**	**LR814264**	**LR814273**	–	**This study**
	*Citrina*	CBS 110.64	Forest soil, Erzurum, Turkey	JN606829	JN606533	MH858377	–	Houbraken et al. 2011
	*Citrina*	CBS 441.88	Sandy soil, Chile	JN606846	JN606568	–	–	Houbraken et al. 2011
	*Citrina*	CBS 643.73	Sandy soil, Manitoba, Canada	–	JN606576	–	–	Houbraken et al. 2011
	*Citrina*	CBS 644.73	Sandy soil, Manitoba, Canada	LR814213	JN606577	–	–	Houbraken et al. 2011
	*Citrina*	CBS 685.85 (Type of *P.lacussarmientei*)	Sandy soil, Torres del Paine National Park, Tierra del Fuego, Chile	JN606855	JN606533	JN617711	–	Houbraken et al. 2011
	*Citrina*	CBS 300.67	Sandy greenhouse soil, The Netherlands	–	JN606561	–	–	Houbraken et al. 2011
	*Citrina*	CBS 127029	Forest soil, Los Alerces National Park, Argentina	JN606814	–	MH864309	–	Houbraken et al. 2011
	*Citrina*	CBS 118024	Ants (*Camponotus* spp.), New Brunswick, Canada	JN606833	JN606537	–	–	Houbraken et al. 2011

CBS: Culture collection of the Westerdijk Fungal Biodiversity Institute, Utrecht, The Netherlands; FMR: Facultat de Medicina i Ciències de la Salut, Reus, Spain. ^T^ Indicate ex-type strains. ^1^ Strain no.: strain number. ^2^*tub*2: β-tubulin; *cmdA*: calmodulin; ITS: Internal transcribed spacer regions of the rDNA and 5.8S region; *rpb*2: the DNA dependent RNA polymerase II largest subunit. Novelties and sequences generated in this study are in bold.

All isolates were preserved and deposited into the fungal collection of the Faculty of Medicine, Reus (FMR). Those isolates that were representative of novel species, ex-type cultures and holotypes (dried cultures) were prepared and deposited at the Westerdijk Fungal Biodiversity Institute (CBS, Utrecht, The Netherlands).

### ﻿DNA extraction, sequencing and phylogenetic analysis

Isolates were cultured on PDA for 7–14 days at 25 °C in darkness. The DNA was extracted through the modified protocol of Werner et al. (1998). Preliminary species identifications were carried out by similarity searches of obtained *tub*2 DNA sequences against reference sequences on GenBank. In the case of putative new species, the ITS region and fragments of *cmdA* and *rpb*2 genes were amplified and sequenced (Visagie et al. 2014b). The primer pairs used for gene amplifications were: Bt2a/Bt2b for *tub*2 (Glass and Donaldson 1995), ITS5/ITS4 for ITS (White et al. 1990), CMD5/CMD6 for *cmdA* (Hong et al. 2006) and RPB2–5F/RPB2–7Cr for *rpb*2 (Liu et al. 1999). The PCR conditions were carried out using primers and methods previously described (Peterson 2008; Houbraken and Samson 2011; Visagie et al. 2014a). Amplified products were purified and sequenced at Macrogen (Madrid, Spain). Consensus sequences were obtained using SeqMan v. 7.0.0 (DNAStar Lasergene, Madison, WI, USA).

Sequences for phylogenies of *Penicillium* sections and series included in the study were retrieved from NCBI GenBank (https://www.ncbi.nlm.nih.gov/genbank/), considering the accepted species of penicillia included in the last update of the International Commission of *Penicillium* and *Aspergillus* database (http://www.aspergilluspenicillium.org), and those more recently published (Houbraken et al. 2020; Visagie et al. 2021). Initially, a preliminary phylogenetic analysis with *tub*2 sequences was carried out to resolve the taxonomic position of our isolates at section level. Thereafter, single and concatenated phylogenetic analyses for each section were calculated to allocate the isolates at series level and establish phylogenetic relationships among closely related species. Individual analyses of the alternative molecular markers of the series in which the isolates under study belong are included as supplementary material.

Datasets for each locus were aligned individually in MEGA (Molecular Evolutionary Genetic Analysis) software v. 6.0. (Tamura et al. 2013) using the CLUSTALW algorithm (Thompson et al. 1994) and refined with MUSCLE (Edgar 2004) or manually adjusted, if necessary, on the same platform. Phylogenetic concordance of the four-locus datasets was evaluated individually through visual comparison of each single-locus phylogeny to assess any incongruent results among nodes with high statistical support. Once the lack of incongruence was confirmed, individual alignments were concatenated into a single data matrix with SequenceMatrix (Vaidya et al. 2011). The best substitution model for all gene matrices was estimated using MEGA software for Maximum Likelihood (ML) analysis, while for the Bayesian Inference (BI) analysis it was estimated using jModelTest v.2.1.3 according to the Akaike criterion (Guindon and Gascuel 2003; Darriba et al. 2012). The phylogenetic reconstructions were made with the combined genes using ML under RAxML-HPC2 on XSEDE v-8.2.12 (Stamatakis 2014) on the CIPRES Science gateway portal (Miller et al. 2012) and BI with MrBayes v.3.2.6 (Ronquist et al. 2012).

For ML, phylogenetic support for internal branches was assessed by 1,000 ML bootstrapped pseudoreplicates and bootstrap support (bs) ≥ 70 was considered significant (Hillis and Bull 1993). The phylogenetic reconstruction by BI was carried out using 5 million Markov chain Monte Carlo (MCMC) generations, with four runs (one cold chain and three heated chains), and samples were stored every 1,000 generations. The 50% majority-rule consensus tree and posterior probability (pp) values were calculated after discarding the first 25% of samples. A pp value of ≥ 0.95 was considered significant (Hespanhol et al. 2019). The resulting trees were plotted using FigTree v.1.3.1 (http://tree.bio.ed.ac.uk/software/figtree/).

The DNA sequences and alignments generated in this study were deposited in GenBank (Table 1) and in TreeBASE under the submission number: 28954 (http://treebase.org), respectively.

### ﻿Phenotypic study

Phenotypic characterization of the strains was made using standard growth conditions established previously (Visagie et al. 2014a). Strains were inoculated in three-point fashion onto Czapek Yeast Autolysate agar (CYA; Pitt 1979), 2% Malt Extract Agar (MEA; Samson et al. 2010), Yeast Extract Sucrose agar (YES; Frisvad 1981), Oatmeal agar (OA; Samson et al. 2010), Dichloran 18% Glycerol agar (DG18; Hocking and Pitt 1980) and Creatine Sucrose agar (CREA; Frisvad 1981) and incubated at 25 °C for 7d in darkness, with the exception of FMR 16948 which was incubated for 14d for sporulation to occur. Colony growth rates were measured after 7 d at 5, 15, 20, 30, 35, 37, and 40 °C on CYA. Color annotations in descriptions follow Kornerup and Wanscher (1978). Microscopic measurements and features were described from colonies grown on MEA after 7 d (14 d for FMR 16948) of incubation. Microscopic slides were mounted with Shear’s solution, after prior removal of the excess conidia with 70% ethanol. Photomicrographs were obtained using a Zeiss Axio-Imager M1 light microscope (Zeiss, Oberkochen, Germany) with a DeltaPix Infinity x digital camera. Photoplates were assembled from separate photographs using PhotoShop CS6.

## ﻿Results

### ﻿Phylogenetic analyses

Among the penicillium-like fungi found, we recovered 15 isolates (12 from PDA at 0.2% cycloheximide; three from DRBC), which preliminary identification, having compared their *tub*2 sequences through BLAST search, revealed as possibly representing putative novel or uncommon species of penicillia (Table 1). According to the *tub*2 phylogeny, *Penicillium* isolates were distributed over two major clades corresponding to the two subgenera of *Penicillium* (i.e. *Aspergilloides* and *Penicillium*) and all sections included in the analysis were well delineated (Suppl. material 1: Fig. S1). Five of the isolates under study, which were distributed in the sections *Robsamsonia* (FMR 17140), *Paradoxa* (FMR 18076), *Canescentia* (FMR 17859), *Lanata-Divaricata* (FMR 16948) and *Gracilenta* (FMR 17747), could not be assigned to any currently accepted species in the genus. Another eight isolates, which belong to the sectionCitrina (FMR 17531, FMR 17534, FMR 17616, FMR 17617, FMR 17619, FMR 17967, FMR 18100 and FMR 18123), were closely related to the ex-type strains of *P.sanguifluum*, *P.roseopurpureum* and *P.vaccaeorum*, the latter species currently considered synonym of *P.sanguifluum* (Houbraken et al. 2011). The other two isolates, FMR 17137 and FMR 18043, were placed in sections *Chrysogena* and *Exilicaulis*, respectively. However, the *tub*2 marker did not show enough discriminatory power to resolve the identity of both isolates concerning their counterparts, which were the ex-type strains of *P.chrysogenum*, *P.tardochrysogenum* and *P.rubens* for FMR 17137, and *P.restrictum* and *P.heteromorphum* for FMR 18043. Phylogenetic analyses and species delimitation were performed using ITS, *tub*2, *cmdA*, and *rpb*2. Dataset characteristics and substitution models for each data partition are summarized in Table 2. Since tree topologies were similar and congruent between the ML and BI analyses in all cases, we selected ML trees to represent section results. Bootstrap support values ≥70% and BI posterior probability values ≥0.95 are indicated on branches.

**Table 2.** Overview and details used for phylogenetic analyses in sections of *Penicillium* analysed in this study.

Dataset		Sections							
		*Chrysogena*	*Robsamsonia*	*Paradoxa/Turbata*	*Canescentia*	*Exilicaulis*	*Lan.-Div.*	*Gracilenta*	*Citrina*
*tub*2	Length (bp)	499	468	493	431	503	547	508	470
*tub*2	Pvar	113	171	173	161	254	307	189	240
*tub*2	Pi	65	103	108	101	205	257	93	207
*tub*2	Model*	K2+G	K2+G	K2+G	K2+G	HKY+G+I	HKY+G+I	HKY+G	HKY+G+I
*tub*2	Model**	K2+G	K2+G	K2+G	K2+G	K2+G	K2+G+I	TN93+G	K2+G
*cmdA*	Length (bp)	531	542	529	560	604	626	642	626
*cmdA*	Pvar	177	202	221	189	302	392	264	378
*cmdA*	Pi	93	124	136	95	349	329	145	337
*cmdA*	Model*	K2+G	K2+I	SYM+G	K2+G	SYM+G+I	HKY+G+I	K2+I	SYM+G+I
*cmdA*	Model**	K2+G	K2+G	K2+I	K2+G	K2+G+I	K2+G+I	K2+I	K2+G+I
ITS	Length (bp)	580	605	572	610	576	559	566	566
ITS	Pvar	25	60	39	45	102	180	44	119
ITS	Pi	7	17	21	33	60	154	20	99
ITS	Model*	K2+G	K2+G	K2+I	K2	K2+G+I	GTR+G+I	GTR+I	GTR+G+I
ITS	Model**	T92	JC+G	K2+I	T92	K2+G+I	GTR+G+I	T92+I	K2+G+I
*rpb*2	Length (bp)	-	936	929	915	950	837	978	-
*rpb*2	Pvar	-	249	256	230	360	345	294	-
*rpb*2	Pi	-	176	183	121	306	313	113	-
*rpb*2	Model*	-	SYM+G	SYM+G	K2+G	SYM+G+I	GTR+G+I	SYM+G	-
*rpb*2	Model**	-	K2+G	K2+I	K2+G	K2+G+I	K2+G+I	TN93+G	-
Concatenated	Length (bp)	1610	2551	2523	2516	2633	2569	2694	1662
Concatenated	Pvar	315	628	689	625	1018	1224	791	737
Concatenated	Pi	165	420	448	350	820	1053	371	643
Concatenated	Model*	K2+G+I	SYM+G	SYM+G	K2+G+I	HKY+G+I	GTR+G+I	GTR+G	SYM+G+I
Concatenated	Model**	K2+G	K2+G	K2+G	K2+G	K2+G+I	GTR+G+I	K2+I	K2+G

Lan.-Div. = sect.Lanata-Divaricata; Pvar = variable sites; Pi = phylogenetic informative sites; *= substitution model for Bayesian inference; **= substitution model for ML analysis; K2 = Kimura 2-parameter; HKY = Hasegawa-Kishino-Yano; SYM = Symmetrical; GTR = General Time Reversible; TN93 = Tamura-Nei; T92 = Tamura 3-parameter; JC = Jukes-Cantor; G = Gamma Distributed; I = Invariant Sites.

**Figure 1.**
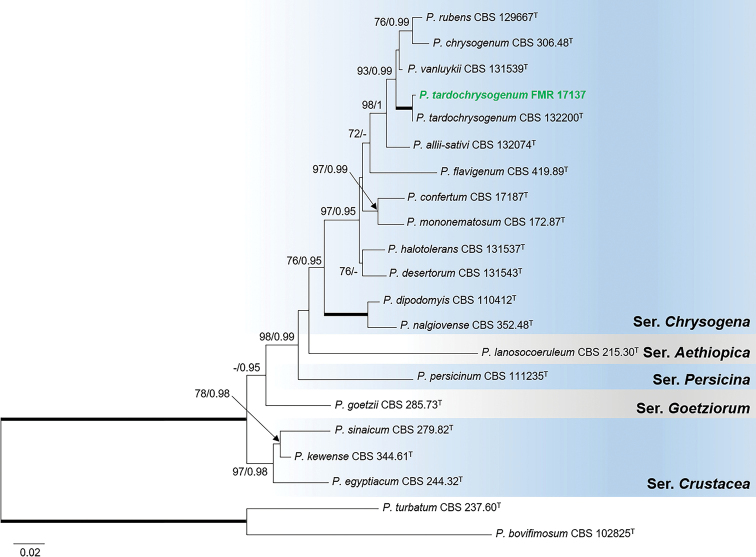
Phylogenetic tree of PenicilliumsectionChrysogena based on ML analysis obtained by RAxML inferred from the combined *tub*2, *cmdA* and ITS loci. Branch lengths are proportional to phylogenetic distance. Bootstrap support values/Bayesian posterior probability scores above 70%/0.95 are indicated on the nodes. Bold branches indicate bs/pp values 100/1. The tree is rooted to *P.turbatum*CBS 237.60 and *P.bovifimosum*CBS 102825. The name in green is the strain of *P.tardochrysogenum* included in this study. ^T^= Ex-type strain.

The concatenate phylogeny for the sectionChrysogena was constructed with ITS, *tub*2 and *cmdA* markers since *rpb*2 was not available for FMR 17137. This isolate was placed in a fully-supported clade (100 bs/1 pp) with the ex-type strain of *P.tardochrysogenum* (Fig. 1). Both specimens showed a high sequence similarity (99.2% for *tub*2, 98.9% *cmdA*, and 99.8% ITS). It is closely related to the type species of the section *P.chrysogenum* and its relatives (*P.rubens*, *P.allii-sativa*, *P.rubens* and *P.vanluytii*). Additional analyses of the series *Chrysogena* with *tub*2 and *cmdA* sequences, including ex-type strains and more reference strains of those mentioned species, confirmed the identity of the sediment isolate (Suppl. material 1: Fig. S2). *Penicilliumtardochrysogenum* has so far been isolated exclusively from soil and rock samples in Antarctica (Houbraken et al. 2012; Alves et al. 2019), and our specimen is thus the first report of the species from temperate regions.

**Figure 2.**
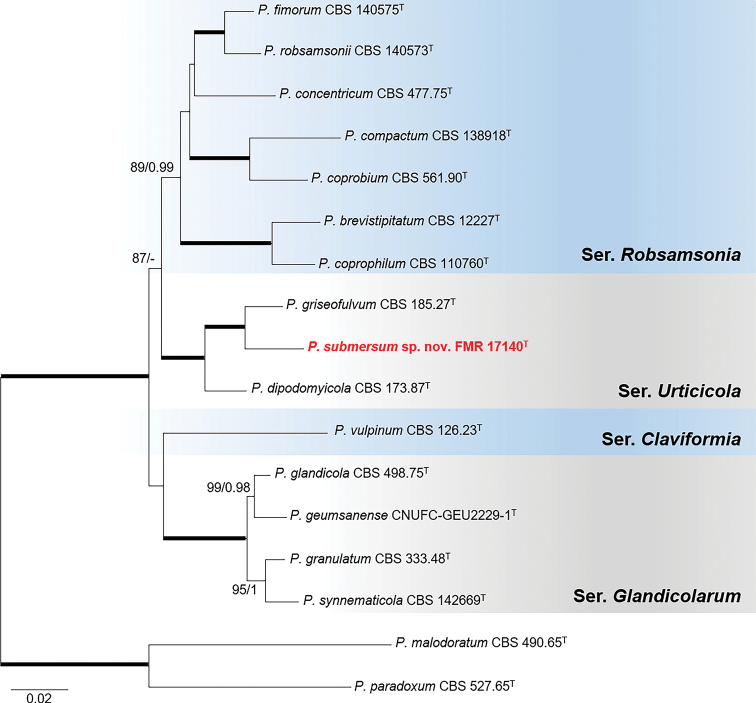
Phylogenetic tree of PenicilliumsectionRobsamsonia based on ML analysis obtained by RAxML inferred from the combined *tub*2, *cmdA*, ITS, and *rpb*2 loci. Branch lengths are proportional to phylogenetic distance. Bootstrap support values/Bayesian posterior probability scores above 70%/0.95 are indicated on the nodes. Bold branches indicate bs/pp values 100/1. The tree is rooted to *P.paradoxum*CBS 527.65 and *P.malodoratum*CBS 490.65. The name in red is the new species described in this study. T= Ex-type strain.

Phylogenetic reconstruction of sectionRobsamsonia with the four markers (Fig. 2) showed the unidentified isolate FMR 17140 grouped with the ex-type strain of *P.griseofulvum* in a fully-supported terminal clade (100 bs/1 pp), but forming an independent and single branch with enough genetic distance (97.0% *tub*2, 97.7% *cmdA*, 99.6% ITS, and 97.2% *rpb*2 similarity) with the ex-type of *P.griseofulvulm* to be considered a distinct phylogenetic species. In order to evaluate possible inter- and intraspecific variability regarding closely related species, we carried out additional analyses with *tub*2, *cmdA* and *rpb*2 markers of the species in series *Urticicola* (Suppl. material 1: Fig. S3), including more sequences of *P.griseofulvum* retrieved from GenBank. These analyses showed that our isolate was always placed distant to the clade representative of *P.griseofulvum*. Therefore, genetic and phenotypic differences, such as a faster growth rate on CYA and strong acid production compared to *P.griseofulvum*, allow us to propose the novel species *Penicilliumsubmersum*.

**Figure 3.**
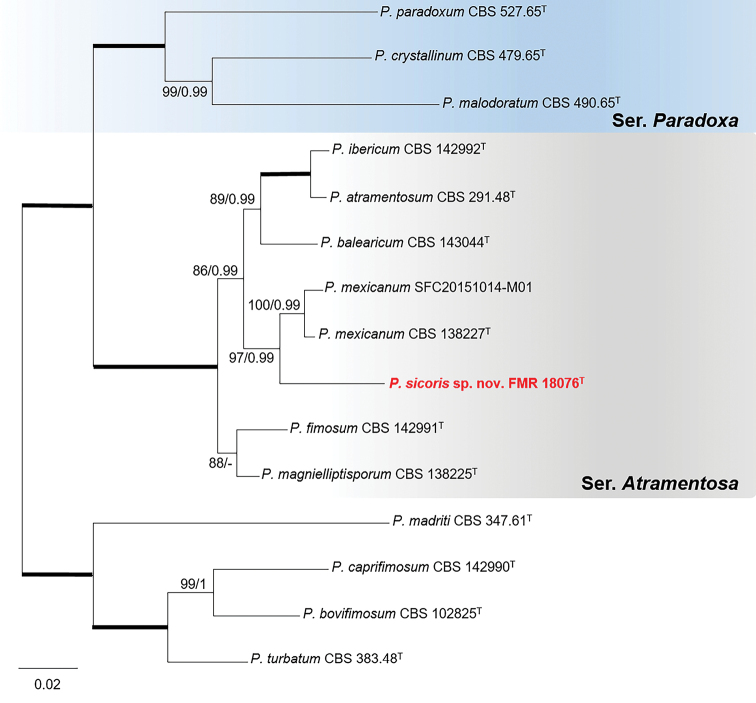
Phylogenetic tree of PenicilliumsectionParadoxa based on ML analysis obtained by RAxML inferred from the combined *tub*2, *cmdA*, ITS, and *rpb*2 loci. Branch lengths are proportional to phylogenetic distance. Bootstrap support values/Bayesian posterior probability scores above 70%/0.95 are indicated on the nodes. Bold branches indicate bs/pp values 100/1. The tree is rooted to Penicillium species belonging to sectionTurbata (*P.madriti*CBS 347.61, *P.caprifimosum*CBS 142990, *P.bovifimosum*CBS 102825 and *P.turbatum*CBS 383.48). The name in red is the new species described in this study. ^T^= Ex-type strain.

The concatenated dataset for sectionParadoxa (Fig. 3) revealed that FMR 18076 belonged to the series *Atramentosa* and was closely related to the lineage representative of *P.mexicanum*, a species recently described from house dust of which only two specimens are known (Visagie et al. 2014c; Park et al. 2019). Both lineages were also well differentiated when additional analyses of the series were carried out with the three alternative markers (*tub*2, *cmdA* and *rpb*2) and more representative sequences of the species in the series (Suppl. material 1: Fig. S4). The genetic distance with the ex-type strain of *P.mexicanum* (96.5%, 94.3% 98.2%, and 97.6% sequence similarity for *tub*2, *cmdA*, ITS, and *rpb*2, respectively) and their morphological differences in colony color, growth rates, and conidial shape (see Taxonomy section) allow to propose FMR 18076 as *Penicilliumsicoris*.

**Figure 4.**
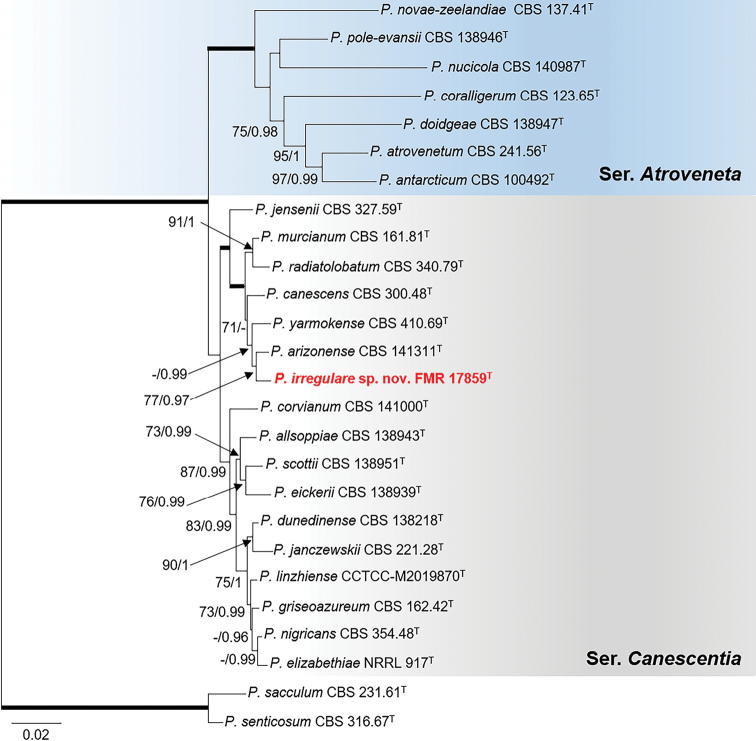
Phylogenetic tree of PenicilliumsectionCanescentia based on ML analysis obtained by RAxML inferred from the combined *tub*2, *cmdA*, ITS, and *rpb*2 loci. Branch lengths are proportional to phylogenetic distance. Bootstrap support values/Bayesian posterior probability scores above 70%/0.95 are indicated on the nodes. Bold branches indicate bs/pp values 100/1. The tree is rooted to *P.sacculum*CBS 231.61 and *P.senticosum*CBS 316.67. The name in red is the new species described in this study. ^T^= Ex-type strain.

The phylogeny constructed for sectionCanescentia (Fig. 4) placed FMR 17859 in a divergent lineage closely related to the ex-type strains of *P.arizonense* and *P.yarmokense*, the three forming a terminal clade only supported with BI analysis (- bs/0.99 pp). In order to elucidate possible inter- and intraspecific variability among these close relatives, additional analyses of the series with the three alternative markers and with more sequences of reference strains of those mentioned species were carried out (Suppl. material 1: Fig. S5). All revealed that *P.arizonense* was the closest relative and FMR 17859 being always placed in a distant branch. Phylogentic and phenotypic differences like stipe and metulae length, its ability to grow at 37 °C and its reverse color on CYA support the proposal of the novel species *Penicilliumirregulare*.

**Figure 5.**
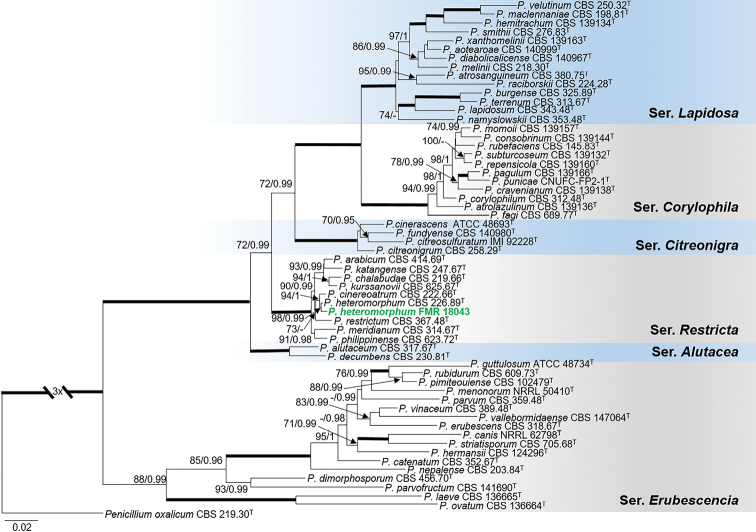
Phylogenetic tree of PenicilliumsectionExilicaulis based on ML analysis obtained by RAxML inferred from the combined *tub*2, *cmdA*, ITS, and *rpb*2 loci. Branch lengths are proportional to phylogenetic distance. Bootstrap support values/Bayesian posterior probability scores above 70%/0.95 are indicated on the nodes. Bold branches indicate bs/pp values 100/1. The tree is rooted to *P.oxalicum*CBS 219.30. The name in green is the strain of *P.heteromorphum* included in this study. ^T^= Ex-type strain.

Identification of FMR 18043 as *P.heteromorphum* was confirmed with the multi-locus phylogeny of sectionExilicaulis (Fig. 5). This species belongs to series *Restricta*, which includes species with still unresolved phylogeny (Visagie et al. 2016). In the tree, our isolate grouped in a strongly supported terminal clade (98 bs/0.99 pp) with the monotypic species *P.heteromorphum* (Kong and Qi 1988). However, despite the high genetic similarity of both strains in *tub*2, *cmdA*, ITS, and *rpb*2 (99.2%, 99.8%, 99.6% and 99.4%, respectively), we observed morphological differences regarding ornamentation of conidia, length of conidiophores and its growth at 37 °C. These differences are described in the Taxonomy section. Additional analyses with more sequences of representative strains of its closely related species in series *Restricta* also supported the identification of our sediment isolate (Suppl. material 1: Fig. S6).

**Figure 6.**
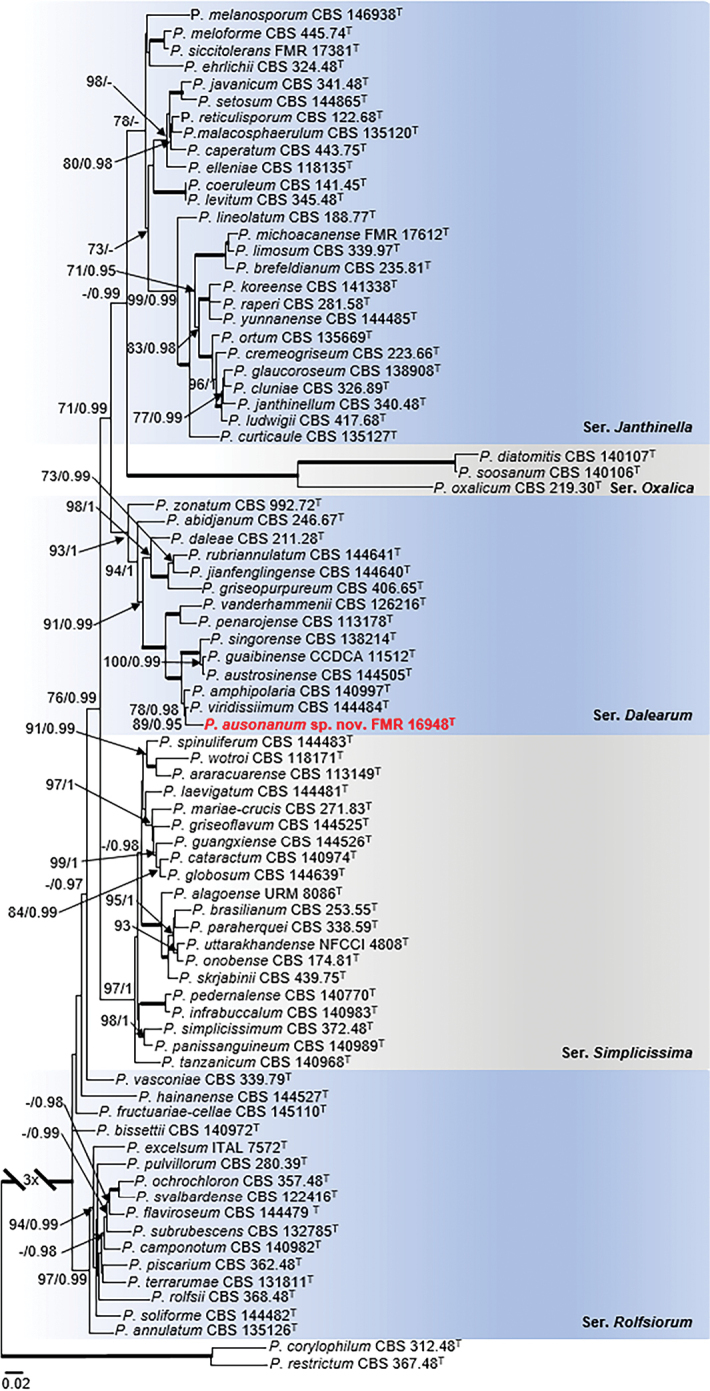
Phylogenetic tree of PenicilliumsectionLanata-Divaricata based on ML analysis obtained by RAxML inferred from the combined *tub*2, *cmdA*, ITS, and *rpb*2 loci. Branch lengths are proportional to phylogenetic distance. Bootstrap support values/Bayesian posterior probability scores above 70%/0.95 are indicated on the nodes. Bold branches indicate bs/pp values 100/1. The tree is rooted to *P.restrictum*CBS 367.48 and *P.corylophilum*CBS 312.48. The name in red is the new species described in this study. ^T^= Ex-type strain.

Phylogenetic reconstruction of sectionLanata-Divaricata (Fig. 6) resolved FMR 16948 in series *Dalearum* closely related to the ex-type strains of *P.amphipolaria* and *P.viridissimum* (78 bs/0.98 pp), but the three specimens were placed in distant lineages. In addition, FMR 16948 showed a similarity of 97.1% *tub*2, 97.2% *cmdA* and 97.4% *rpb*2 with the ex-type strain of *P.amphipolaria* and of 97.8% *tub*2, 97.7% *cmdA* and 98.4% *rpb*2 with the ex-type of *P.viridissimum*. Similar results were obtained in analyses of the series with alternative markers and more sequences of representative strains of its closest relatives (Suppl. material 1: Fig. S7). Our isolate also differed from its relatives by strong acid production and by the predominance of mono- and biverticillate conidiophores. Therefore, morphological and phylogenetic differences support to propose our isolate as *Penicilliumausonanum*.

**Figure 7.**
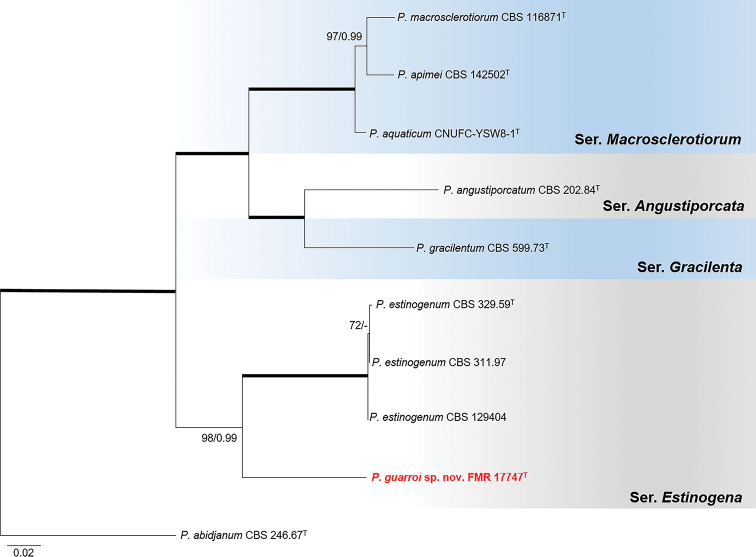
Phylogenetic tree of PenicilliumsectionGracilenta based on ML analysis obtained by RAxML inferred from the combined *tub*2, *cmdA*, ITS, and *rpb*2 loci. Branch lengths are proportional to phylogenetic distance. Bootstrap support values/Bayesian posterior probability scores above 70%/0.95 are indicated on the nodes. Bold branches indicate bs/pp values 100/1. The tree is rooted to *P.abidjanum*CBS 246.67. The name in red is the new species described in this study. ^T^ = Ex-type strain.

The phylogenetic analysis of sectionGracilenta (Fig. 7), comprising the six species *P.aquaticum*, *P.angustiporcatum*, *P.apimei*, *P.estinogenum*, *P.gracilentum* and *P.macroesclerotiorum* with considerable genetic distance between them, revealed that FMR 17747 did not belong to any of the lineages of these known species and formed a basal and distant branch neighboring the fully-supported clade representative of *P.estinogenum*. Similarity values of the sediment isolate with respect to the ex-type strain of this latter species were 86.5% for *tub*2, 82.5% for *cmdA*, and 96.8% for ITS. *Rpb2* sequences of *P.estinogenum* were not available for comparison. Individual analyses of the alternative gene markers including sequences of all reference strains available for the species in the section are shown in Suppl. material 1: Fig. S8. This undescribed monophyletic lineage is proposed as *Penicilliumguarroi*, which differed morphologically from its counterpart mainly by its smooth-walled globose conidia.

**Figure 8.**
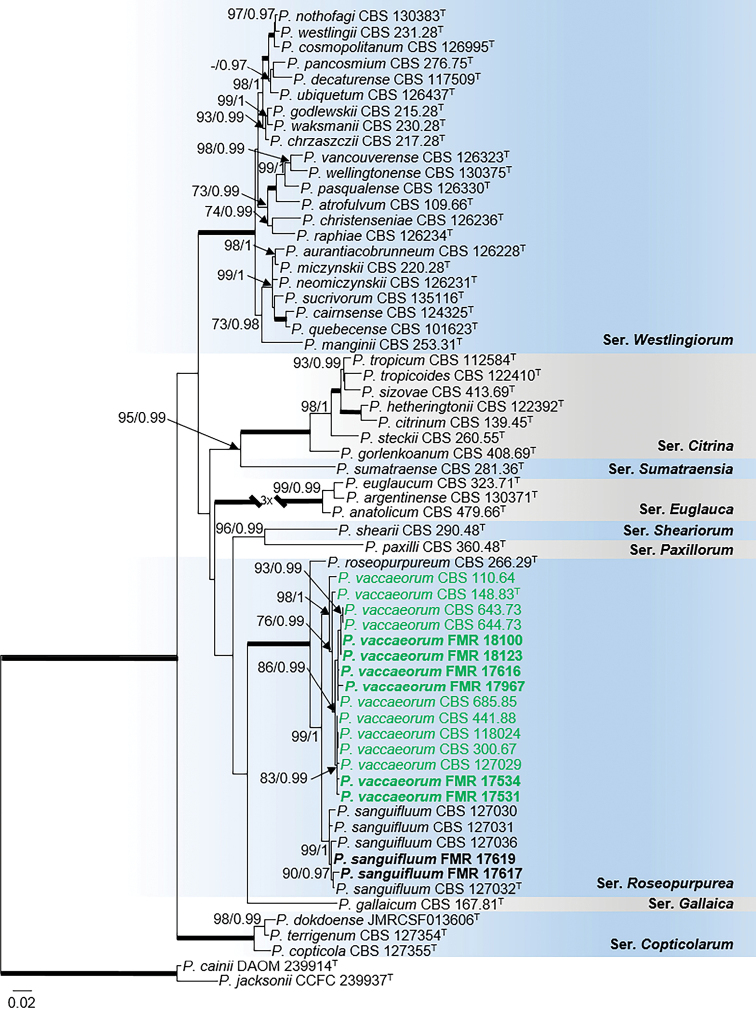
Phylogenetic tree of PenicilliumsectionCitrina based on ML analysis obtained by RAxML inferred from the combined *tub*2, *cmdA*, and ITS loci. Branch lengths are proportional to phylogenetic distance. Bootstrap support values/Bayesian posterior probability scores above 70%/0.95 are indicated on the nodes. Bold branches indicate bs/pp values 100/1. The tree is rooted to *P.cainii* DAOM 239914 and *P.jacksonii* CCFC 239937. The name in green is the resurrected species *P.vaccaeorum* included in this study. ^T^= Ex-type strain.

The concatenated alignment for sectionCitrina (Fig. 8) was carried out with *tub*2, *cmdA* and ITS loci, since sequences of the *rpb2* marker were not available for many species in the section. This multi-locus analysis placed the sediment isolates (FMR 17531, FMR 17534, FMR 17616, FMR 17967, FMR 18100 and FMR 18123) in the series *Roseopurpurea* and grouped them in a well-supported clade (98 bs/1 pp) with the ex-type strains of *P.vaccaeorum* (CBS 148.83) and *P.lacussarmientei* (CBS 685.85), together with other strains (CBS 110.64, CBS 127029, CBS 441.88, CBS 300.67, CBS 118024, CBS 644.73, CBS 643.73) from soil and insects of different countries, and previously considered as *P.sanguifluum* by Houbraken et al. (2011). This terminal clade was sister to the also strongly supported clade with the ex-type strain of *P.sanguifluum* (CBS 127032) and other reference strains from different origins, including some collected in our survey from Spanish fluvial sediments (FMR 17617 and 17619). Both monophyletic lineages showed a genetic distance of 3.4%, which supports considering them distinct species. This result was corroborated with additional analyses of the alternative markers *tub*2 and *cmdA*, including more sequences of reference strains for the species in the series (Suppl. material 1: Fig. S9). In addition, features such as faster growth on YES agar, the ability to grow at 35 °C and longer stipes, allow us to distinguish the clade represented by *P.vaccaeorum* from that of *P.sanguifluum*. Since we accept *P.vaccaeorum* as a distinct species, a detailed description is provided below.

## ﻿Taxonomy

### ﻿ Penicillium ausonanum

Taxon classificationFungiEurotialesAspergillaceae

Torres-Garcia, Gené and Dania García sp. nov.

840556

Figure 9

#### Etymology.

Referring to *Ausona* (Osona), the region of Catalonia where the fungus was collected.

#### Type.

Spain, Catalonia, Osona, Guilleries-Savassona Natural Park, Malafogassa, Major Stream, from sediments, Nov. 2018, *E. Carvalho* & *J. Gené* (***holotype***CBS H-24781, cultures ex-type CBS 148237 = FMR 16948).

**Figure 9.**
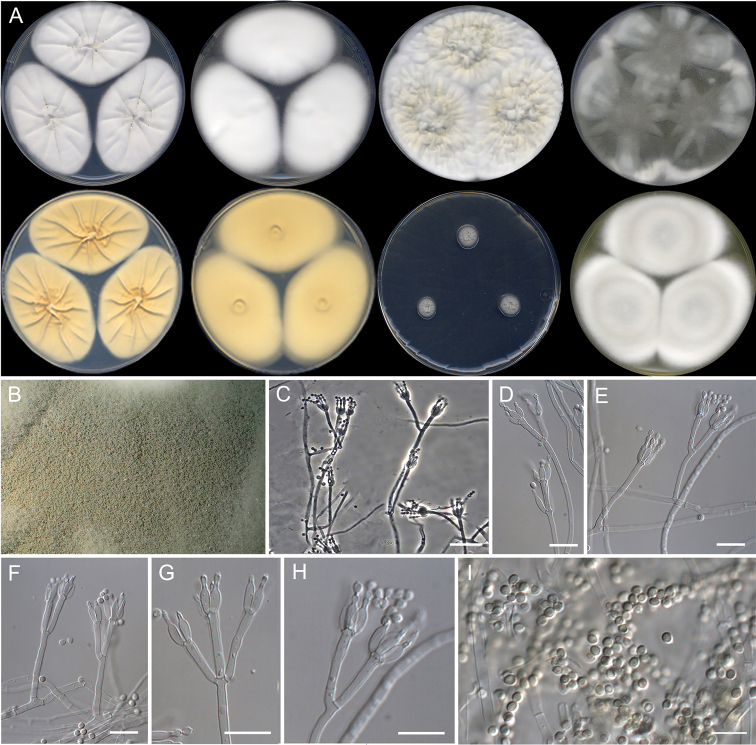
Morphological characters of *Penicilliumausonanum* sp. nov. (ex-type FMR 16948). **A** colonies from left to right (top row) CYA, MEA, YES, and OA; (bottom row) CYA reverse, MEA reverse, DG18, and CREA**B** colony on OA under stereoscopic magnifying glass **C–H** conidiophores **I** conidia. Scale Bars: 25 μm (**C**), 10 μm (**D–I**).

#### Subgeneric classification.

SubgenusAspergilloides, sectionLanata-Divaricata, series *Dalearum*.

#### Description.

***Mycelium*** superficial and immersed, composed of septate, smooth-walled, hyaline hyphae, 2–3 μm wide. ***Conidiophores*** monoverticillate and divaricate, minor proportion biverticillate; ***stipes*** smooth-walled, 20–120 × 2–2.5 μm; ***metulae*** slightly appressed to divergent, mostly 2, occasionally 3 per stipe, 10–18 × 2–3 μm, occasionally a solitary phialide borne on the same level as metulae; ***phialides*** 2–5 per stipe/metula, ampulliform to cylindrical, 9–12 × 2–3 μm; ***conidia*** smooth-walled, globose to subglobose, 2–3 × 2–3 μm.

#### Culture characteristics (14 d at 25 °C).

Colonies on CYA, 58–59 mm diam., slightly radially and concentrically sulcate, velvety to floccose, whitish (5A1), margins regular and slightly fimbriate, sporulation absent to sparse, when present conidial masses brownish gray (6C2); reverse grayish yellow (4B4); little production of exudates hyaline, soluble pigment absent. On MEA, 61–62 mm diam., slightly raised, floccose, white (3A1), margins fimbriate, sporulation absent to sparse, conidial masses brownish gray (5E3); reverse light yellow (4A4); exudate absent, soluble pigment absent. On YES, 67–71 mm diam., slightly raised, radially sulcate, randomly furrowed as well, velvety to floccose, dull yellow (3B3) at center and white (3B1) towards periphery, margins slightly fimbriate, sporulation absent to sparse, conidial masses grayish to dull green (25C4–5C); reverse brownish yellow (5C8), exudates and soluble pigment absent. On OA, 63–65 mm diam., slightly raised, white (3A1) with gray (3E1) to olive (3F3) areas, velvety, margins slightly fimbriate, sporulation abundant, conidial masses grayish to dull green (25C5–D5); reverse grayish-yellow (3B5); exudates and soluble pigment absent. On DG18, 10–12 mm diam., randomly furrowed at the center, radially sulcate towards periphery, velvety, yellowish gray (2B2), margins entire, sporulation absent to sparse, conidial masses grayish to dull green (25C4–5C); reverse grayish yellow (2C3); exudates and soluble pigment absent. On CREA, 61–63 mm diam., slightly raised, floccose, gray (4B1) at center to yellowish gray (4B2) and white (4A1) towards periphery, margins slightly fimbriate, sporulation abundant, conidial masses brownish gray (6C2); reverse vivid yellow (3A8); exudates absent, acid production strong.

#### Colony diameter on CYA after 7d (mm).

5 °C 3–2, 15 °C 41–43, 20 °C 46–48, 30 °C 56–57, 35 °C 50–51, 37 °C 38–39, 40 °C no growth.

#### Distribution.

Spain.

#### Notes.

*Penicilliumausonanum* formed a phylogenetically supported group together with *P.amphipolaria* and *P.viridissimum* in series *Dalearum* (Fig. 6). These are two species recently described, the former from soil in Antarctica and Canada, and the latter from acidic and forest soil from China (Visagie et al. 2016a, Diao et al. 2019). The new species can be morphologically differentiated from them by its equal proportion of monoverticillate and divaricate conidiophores, which are mostly with a complex branching pattern in *P.amphipolaria* (biverticillate and divaricate) (Visagie et al. 2016a), and mono- to terverticillate in *P.viridissimum* (Diao et al. 2019). Both *P.amphipolaria* (6.5–10 µm) and *P.viridissimum* (6.5–10 µm) have slightly shorter phialides than *P.ausonanum* (9–12 µm). Also, *P.amphipolaria* (240–460 µm) and *P.viridissimum* (40–125 µm) have longer stipes (Visagie et al. 2016a, Diao et al. 2019) in comparison to those of *P.ausonanum* (20–120 µm). Furthermore, the three species also differed in acid production on CREA, which is strong in *P.ausonanum*, moderate in *P.amphipolaria* and absent in the neighboring species *P.viridissimum*.

### ﻿ Penicillium guarroi

Taxon classificationFungiEurotialesAspergillaceae

Torres-Garcia, Gené and Dania García sp. nov.

840567

Figure 10

#### Etymology.

Named in honor of Josep Guarro for his contributions to our knowledge of microfungi.

#### Type.

Spain, Catalonia, Alt Camp, Alcover, Brugent River, sediments, Mar. 2019, *D. Torres* & *J. Gené* (***holotype***CBS H-24782, cultures ex-type CBS 148238 = FMR 17747).

**Figure 10.**
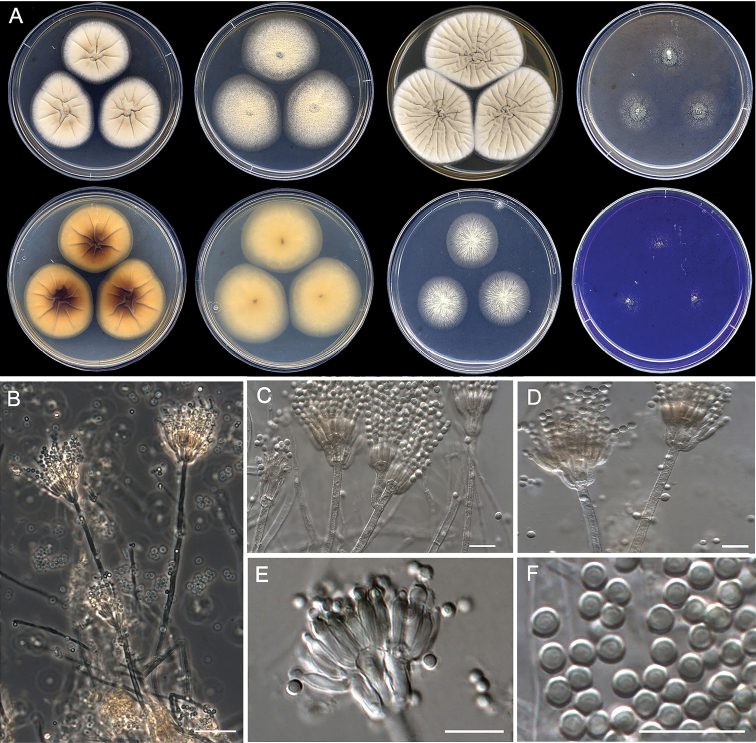
Morphological characters of *Penicilliumguarroi* sp. nov. (ex-type FMR 17747). **A** colonies from left to right (top row) CYA, MEA, YES, and OA; (bottom row) CYA reverse, MEA reverse, DG18, and CREA**B–E** conidiophores on MEA**F** conidia. Scale Bars: 25 μm (**B**), 10 μm (**C–F**).

#### Subgeneric classification.

Subgenus Aspergilloides, sectionGracilenta, series *Estinogena*.

#### Description.

***Mycelium*** superficial and immersed, composed of septate, smooth-walled, hyaline hyphae, 2.5–3.5 μm wide. ***Conidiophores*** predominantly symmetrically biverticillate, occasionally with subterminal branches; ***stipes*** smooth- to rough-walled, 88–215 × 3–4 μm; ***metulae*** appressed, 2–4 per stipe, vesiculate, 5–10 × 2–4.5 μm (vesicle up to 5.5 μm wide); ***phialides*** 3–6 per metula, ampulliform, 6–9 × 1.5–3 μm; ***conidia*** smooth-walled, globose, 2–2.5 × 2–2.5 μm.

#### Culture characteristics (7 d at 25 °C).

Colonies on CYA, 38–40 mm diam., slightly raised at center, radially sulcate, velvety, brownish gray (6C2) and white (1A1) towards periphery, margins fimbriate, sporulation moderate, conidial masses greenish gray (28C2); reverse dark brown (6F6) and light brown (6D6) at periphery, becoming entirely brown after 14 d; soluble pigment absent. On MEA, 41–43 mm diam., slightly raised, granular, yellowish green (29B7) and white (1A1) towards periphery, margins slightly fimbriate, sporulation moderate, conidial masses grayish green (28D2); reverse yellowish brown (5E6) at center to grayish yellow at periphery; soluble pigment absent. On YES, 49–51 mm diam., raised at center, radially sulcate, velvety, brownish gray (5C2) and white (1A1) at periphery, margins entire, sporulation sparse, conidial masses greenish gray (28D2); reverse dark green (30F5) and yellowish brown (5D5) towards periphery; soluble pigment absent. On OA, 24–26 mm diam., elevated at center, velvety, white (1A1) at center and dull green (25E3) towards periphery, margins regular, sporulation moderate, conidial masses dull green (25D4); reverse brown (6E4) and yellowish gray (4B2) at periphery; soluble pigment absent. On DG18, 22–25 mm diam., flattened, granular, grayish green (30C3) at center, and dull green (29D49) towards periphery, margins fimbriate, sporulation moderate, conidial masses greenish gray (27D2); reverse grayish green (30E5) and white (1A1) at periphery, soluble pigment absent. On CREA, 22–25 mm diam., flattened, floccose, yellowish green (29B7) and white (1A1) at periphery, margins irregular, sporulation moderate, conidial masses grayish green (27B3–D3); reverse dark gray (1F1); soluble pigment absent, no acid production.

#### Colony diameter on CYA after 7d (mm).

5 °C no growth, 15 °C 17–19, 20 °C 26–28, 30 °C 34–36, 35 °C 4–5, 37 °C no growth, 40 °C no growth.

#### Distribution.

Spain.

#### Notes.

*Penicilliumguarroi* is the second species included in sectionGracilenta series *Estinogena* (Fig. 7). This species shows morphological attributes of the series based on its type *P.estinogenum*, namely that both have symmetrically appressed biverticillate conidiophores with rough-walled stipes (Houbraken et al. 2020). However, *P.guarroi* mainly differs from *P.estinogenum* by producing strictly smooth-walled globose conidia, which are ellipsoidal to ovate and with smooth to finely roughened walls in the latter (Abe 1956; Houbraken et al. 2020). In addition to their phylogenetic distance, other members of sectionGracilenta (i.e., series *Gracilenta* and *Macrosclerotiorum*) can be differentiated morphologically by the production of monoverticillate conidiophores and the lack of growth at 37 °C, with the exception of *P.apimei* and *P.aquaticum*, which are able to grow at this temperature (Barbosa et al. 2018; Houbraken et al. 2020). *Penicilliumguarroi* was unable to grow at 37 °C, but it shows a maximum temperature for growth at 35 °C (4–5 mm), like other members in the section (i.e., *P.macrosclerotiorum*, *P.angustiporcatum*, *P.gracilentum* and *P.estinogenum*).

### ﻿ Penicillium heteromorphum

Taxon classificationFungiEurotialesAspergillaceae

H.Z. Kong and Z.T. Qi. Mycosystema 1:107. 1988.

Figure 11

#### Subgeneric classification.

Subgenus Aspergilloides, sectionExilicaulis. series *Restricta*.

#### Description.

***Mycelium*** superficial and immersed, composed of septate, smooth-walled, hyaline hyphae, 1.5–3 μm wide. ***Conidiophores*** monoverticillate, occasionally irregularly branched; ***stipes*** smooth-walled, thin, 6–47.5 × 1.5–2 μm; ***phialides*** 2–3 per stipe, ampulliform, 3–7 × 1.5–2.5 μm; ***conidia*** roughened, globose to subglobose, 2.5–3 × 2.5–3 μm, occasionally conidia up to 5 μm were observed.

**Figure 11.**
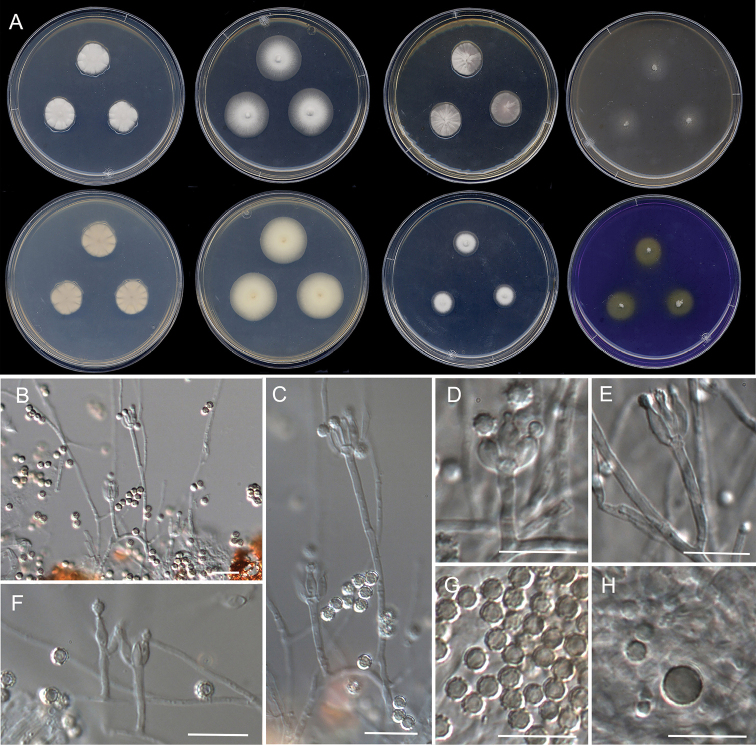
Morphological characters of *Penicilliumheteromorphum* (FMR 18043). **A** colonies from left to right (top row) CYA, MEA, YES, and OA; (bottom row) CYA reverse, MEA reverse, DG18, and CREA**B, C, F** conidiophores on OA. **D, E** conidiophores on Czapek’s Agar **G–H** conidia. Scale Bars: 25 μm (**B**), 10 μm (**C–H**).

#### Culture characteristics (7 d at 25 °C).

Colonies on CYA, 19–20 mm diam., slightly raised at center, velvety, radially sulcate, yellowish gray (4B2) at center and white (1A1) at periphery, margins slightly undulate, sporulation absent; reverse pale yellow (4A3); soluble pigment absent. On MEA, 27–28 mm diam., slightly raised at center, velvety, white (1A1) at center, ash blond (3C3) towards periphery, margins entire, sporulation absent; reverse champagne colored (4A4) at center, pastel yellow (3A4) towards periphery; soluble pigment absent. On YES, 22–21 mm diam., slightly raised at center, velvety, radially sulcate, yellowish gray (4B2) and Sahara colored (6C5), margins entire, sporulation absent; reverse grayish orange (5B5) at center and champagne colored (4B4) towards periphery; soluble pigment absent. On OA, 23–24 mm diam., slightly elevated at center, floccose, greenish gray (28B2) at center and beige (4C3) towards periphery, margins fimbriate, sporulation abundant, conidial masses dull green (29E3); reverse beige (4C3); soluble pigment absent. On DG18, 14–15 mm diam., slightly raised at center, velvety, yellowish white (1A2) at center and white (1A1) towards periphery, margins regular, sporulation absent; reverse wine yellow (3B3) at center and yellowish white (3A2) towards periphery; soluble pigment absent. On CREA reaching 17–19 mm diam., slightly raised at center, floccose, white (1A1) at center and lemon yellow (3B8) towards periphery, margins fimbriate, sporulation absent; reverse lemon yellow (3B8); soluble pigment absent and acid production moderate. Colonies on Czapek’s agar reaching 13–14 mm diam., flattened, floccose, white (1A1) at center to ash gray (1B2) towards periphery, margins entire, sporulation abundant, conidial masses dull green (29D3); reverse ash gray (1B2); soluble pigment absent.

#### Colony diameter on CYA after 7d (mm).

5 °C no growth, 15 °C 9–11, 20 °C 12–13, 30 °C 23–24, 35 °C 16–19, 37 °C 4–7, 40 °C no growth.

#### Specimen examined.

Spain, Catalonia, Berguedà, Gósol, from stream sediments, Nov. 2019, *J. Gené* (CBS 148239, FMR 18043).

#### Distribution.

China and Spain.

#### Notes.

*Penicilliumheteromorphum* was first described from a soil sample collected in Shennongjia, China. FMR 18043 is thus the second isolate of this species. The protologue of *P.heteromorphum*, which was based on CYA and Czapek’s agar, noted that it does not grow at 37 °C, has strictly monoverticillate conidiophores with stipes up to 60 μm long, and produce conidia that are globose to subglobose, smooth or nearly, which show two well-differentiated measures on Czapek’s agar (ones of 2–2.5 (–3) μm diam, and the largest of 4–10 μm) (Kong and Qi 1988). By contrast, despite the high sequence similarity to the ex-type strain, our isolate differs phenotypically in its ability to grow at 37 °C, and in the production of shorter conidiophores and rough-walled conidia in all media studied; some larger conidia (up to 5 μm diam.) were only observed on Czapek’s agar. Nevertheless, features we observed in the sediment isolate *P.heteromorphum* match those of the species of series *Restricta*, which briefly consisted in growing restricted to moderately fast, producing generally short monoverticillate conidiophores with smooth stipes, globose to subglobose or (broadly) ellipsoidal, smooth or roughened conidia and they commonly grow at 37 °C (Houbraken et al. 2020). Based on the production of two types of conidia, Kong and Qi (1988) compared *P.heteromorphum* with *P.dimorphosporum*. However, although both species belongs in sectionExilicaulis, the current taxonomy of the genus places *P.dimorphosporum* in the genetically distant series *Erubescentia* (Visage et al. 2016b, Houbraken et al. 2020).

### ﻿ Penicillium irregulare

Taxon classificationFungiEurotialesAspergillaceae

Torres-Garcia, Gené and Dania García sp. nov.

840558

Figure 12

#### Etymology.

Referring to the variable branching pattern of the conidiophores of the species.

#### Type.

Spain, Comunidad de Madrid, Miraflores de la Sierra, Miraflores River, from sediments, Jun. 2019, *J.F. Cano* (***holotype***CBS H-24783, cultures ex-type CBS 148240 = FMR 17859).

**Figure 12.**
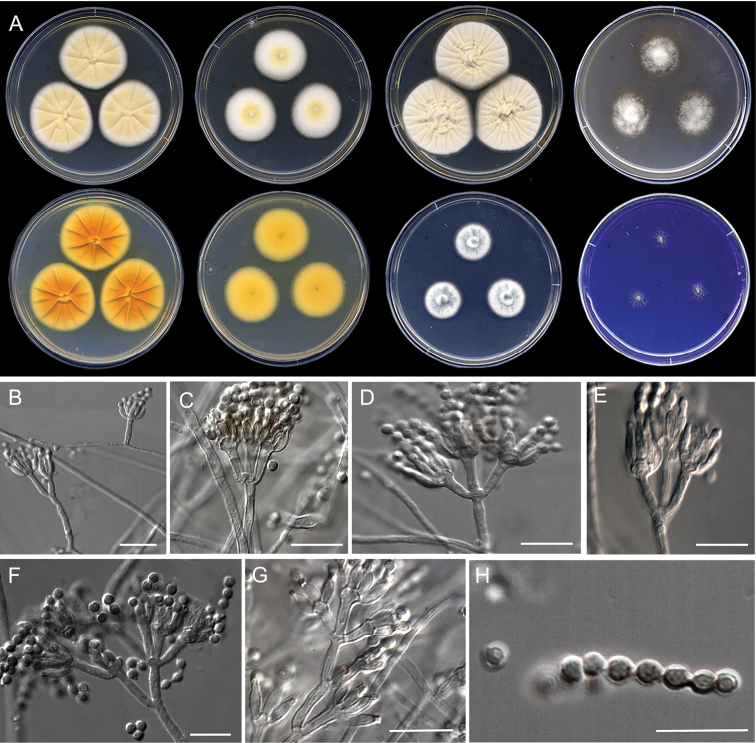
Morphological characters of *Penicilliumirregulare* sp. nov. (ex-type FMR 17859). **A** colonies from left to right (top row) CYA, MEA, YES, and OA; (bottom row) CYA reverse, MEA reverse, DG18, and CREA**B–G** conidiophores on MEA**H** conidia. Scale Bars: 10 μm (**B–H**).

#### Subgeneric classification.

Subgenus Penicillium, sectionCanescentia, series *Canescentia*.

#### Description.

***Mycelium*** superficial and immersed, composed of septate, smooth-walled, hyaline hyphae, 2–3.5 μm wide. ***Conidiophores*** biverticillate, in minor proportion monoverticillate, terverticillate or divaricate; ***stipes*** smooth-walled, 13–152 × 1.5–2 μm; ***metulae*** divergent, 2–3 per stipe/branch, unequal in length, vesiculate, 7–10 × 1.5–2.5 μm (vesicle up to 4 μm wide), occasionally a solitary phialide borne on same level as metulae; ***phialides*** 5–8 per metula, ampulliform, 6–7.5 × 1.5–2.5 μm; ***conidia*** smooth- to finely rough-walled, globose to subglobose, somewhat ellipsoidal, 1.5–3 × 1.5–2 μm.

#### Culture characteristics (7 d at 25 °C).

Colonies on CYA, 36–38 mm diam, slightly elevated, radially sulcate, velvety, grayish orange (6B3) at center and white (1A1) towards periphery, margins entire, sporulation abundant, conidial masses grayish green (25B3); reverse brownish yellow (5C8) at center and vivid yellow towards periphery (3A8); exudate and soluble pigment absent. On MEA, 33–34 mm diam, elevated, floccose, light yellow (4A4) at center and white (1A1) towards periphery, margins fimbriate, sporulation abundant, conidial masses grayish green (25C3); reverse yellowish orange (4A7); exudate and soluble pigment absent. On YES, 43–44 mm diam, raised, concentrically sulcate and pale yellow (4A3) at center, radially sulcate and white (1A1) towards periphery, velvety, margins entire, sporulation absent to sparse, conidial masses grayish green (25D2); reverse brownish yellow (5C8) and white (1A1) at periphery; exudate and soluble pigment absent. On OA, 25–27 mm, slightly elevated at center, cottony and fasciculate, dull green (25E4) at center, gray (1D1) and white (1A1) towards periphery, margins entire, sporulation abundant, conidial masses dull green (30E3); reverse yellowish brown (5F4), golden brown (5D7) towards periphery; soluble pigment absent. On DG18, 14–15 mm, elevated, velvety, white (1A1) with grayish turquoise (24E4) areas, margins slightly fimbriate, sporulation abundant, conidial masses grayish turquoise (24B3–C5); reverse yellowish green (30B8) at center to pale green (30A3) and white (1A1) at periphery; soluble pigment absent. On CREA, 12–13 mm, flat, floccose, yellowish green (29B7), margins regular, sporulation sparse, conidial masses grayish green (27B3–C3); reverse dark gray (1F1); soluble pigment and acid production absent.

#### Colony diameter on CYA after 7d (mm).

5 °C no growth, 15 °C 19–20, 20 °C 25–27, 30 °C 31–33, 35 °C 11–12, 37 °C 5–10, 40 °C no growth.

#### Distribution.

Spain.

#### Notes.

*Penicilliumirregulare* is related to *P.arizonense*, *P.yarmokense* and *P.canescens*, all belonging to series *Canescentia* (Fig. 4). Species of this series are characterized by the production of biverticillate conidiophores, that occasionally produce additional branching stages (divaricate), with smooth- or rough-walled stipes (Houbraken et al. 2020; Visage et al. 2021). *Penicilliumirregulare* can be differentiated from its closest relative *P.arizonense* by the production of shorter stipes (13–152 μm vs 50–400 μm) and metulae (7–10 μm vs. 8–16 μm), and colony reverse yellowish to orange, in contrast to brown shades, even red brown to violet brown on YES agar in *P.arizonense* (Grijseels et al. 2016). In addition, *P.irregulare* was able to grow at 37°C on CYA, but restrictedly (5–10 mm diam. 7 d), while *P.arizonense* does not grow at this temperature.

### ﻿ Penicillium sicoris

Taxon classificationFungiEurotialesAspergillaceae

Torres-Garcia, Gené and Dania García sp. nov.

840559

Figure 13

#### Etymology.

Referring to the Segre River where the fungus was found.

#### Type.

Spain, Catalonia, La Noguera, Camarassa, Segre river, from sediments, Dec. 2019, *D. Torres* & *J. Gené* (***holotype***CBS H-24784, cultures ex-type CBS 148241 = FMR 18076).

**Figure 13.**
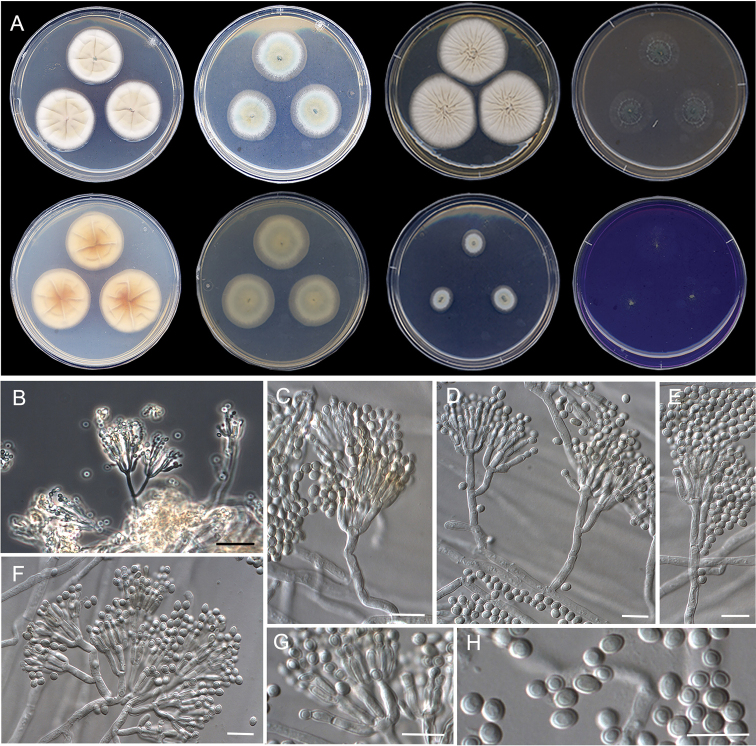
Morphological characters of *Penicilliumsicoris* sp. nov. (ex-type FMR 18076). **A** colonies from left to right (top row) CYA, MEA, YES, and OA; (bottom row) CYA reverse, MEA reverse, DG18, and CREA**B–G** conidiophores on MEA**H** conidia. Scale Bars: 25 μm (**B**), 10 μm (**C–H**).

#### Subgeneric classification.

Subgenus Penicillium, sectionParadoxa, series *Atramentosa*.

#### Description.

***Mycelium*** superficial and immersed, composed of septate, smooth-walled, hyaline hyphae, 3–5 μm wide. ***Conidiophores*** biverticillate or terverticilliate, occasionally irregularly branched with phialides growing directly from branches and divaricate; ***stipes*** smooth-walled, 25–215 × 3–4.5 μm; ***metulae*** divergent, 2–3 per branch, vesiculate, 7–20 × 2.5–4 μm (vesicle up to 5.5 μm wide); ***phialides*** 1–6 per metula, ampulliform, 4–7.5 × 2.5–4 μm; ***conidia*** smooth-walled, usually globose to subglobose, some broadly ellipsoidal, 2–4.5 × 2–3.5 μm.

#### Culture characteristics (7 d at 25 °C).

Colonies on CYA, 32–34 mm diam., raised at center, radially sulcate, velvety, brownish violet (11D8) at center, pale orange (5A3) and white (1A1) towards periphery, margins entire, sporulation abundant, conidial masses grayish green (28B3); reverse light orange (6A5) to orange (6B7) at center and grayish yellow (4B4) towards periphery; soluble pigment absent. On MEA, 28–30 mm diam., flat, velvety, grayish green (30D6) at center, bluish green (25C8), and white (1A1) at periphery, margins entire, sporulation abundant, conidial masses grayish turquoise (24C3–C4); reverse pea green (29D5), yellowish white (4A2); soluble pigment absent. On YES, 39–43 mm diam., raised at center, radially sulcate, velvety, orange gray (5B2) at center and white (1A1) towards periphery, margins entire, sporulation sparse, conidial masses grayish green (28C3); reverse grayish yellow (4B4) and pale yellow (4A3) at periphery; soluble pigment absent. On OA, 23–24 mm diam., slightly elevated at center, floccose, grayish green (26E6), opaline green (25C6) and brownish gray (5C2) towards periphery, margins slightly fimbriate, sporulation abundant, conidial masses dull green (27D3); reverse pea green (29D5) at center and brownish gray (5C2) towards periphery; soluble pigment absent. On DG18, 13–16 mm diam., slightly raised at center, velvety, olive (3D3) at center, grayish turquoise (24B3) and white (1A1) towards periphery, margins entire, sporulation abundant, conidial masses grayish turquoise (24B3); reverse, grayish green (1C4) and white (1A1) at periphery; soluble pigment absent. On CREA, 21–27 mm diam., slightly elevated at center, velutinous, apple green (29C7), margins regular, sporulation abundant, conidial masses grayish green (26B3–C3); reverse colorless; soluble pigment absent, acid production absent.

#### Colony diameter on CYA after 7d (mm).

5 °C 3–4, 15 °C 25–26, 20 °C 30–31, 30 °C 29–31, 35 °C no growth, 37 °C no growth, 40 °C no growth.

#### Distribution.

Spain.

#### Notes.

*Penicilliumsicoris* is closely related to *P.mexicanum* in series *Atramentosa* (Fig. 3). Phenotypically, species of this series share a moderately fast colony growth and brown reverse on CYA and YES, and good growth on CREA without acid production (Houbraken et al. 2020). However, our species differs in having an orange to grayish yellow reverse on CYA. In addition, *P.sicoris* also differs from its counterpart in several micromorphological features: i.e., its conidiophores are bi- or terverticillate, whereas in *P.mexicanum* they are ter- or quaterverticillate, stipes are shorter (25–215 vs. 65–370 μm), phialides shorter (4–7.5 vs. 7–10 μm) and metulae longer (7–20 vs. 8.5–15.5 μm) than those of *P.mexicanum*, and its conidia are predominantly globose to subglobose, whereas in *P.mexicanum* they are broadly ellipsoidal to ellipsoidal (Visagie et al. 2014c). Moreover, *P.mexicanum* has a more restrictive growth on CREA than *P.sicoris* (5–8 vs. 21–27 mm diam. after 7 d).

### ﻿ Penicillium submersum

Taxon classificationFungiEurotialesAspergillaceae

Torres-Garcia, Gené and Dania García sp. nov.

840560

Figure 14

#### Etymology.

Referring to the submerged sediment sample where the fungus was isolated.

#### Type.

Spain, Catalonia, Montsant Natural Park, Siurana’s Swamp, from sediments, Feb. 2018, *E. Carvalho* & *J. Gené* (***holotype***CBS H-24785, cultures ex-type CBS 148242 = FMR 17140).

**Figure 14.**
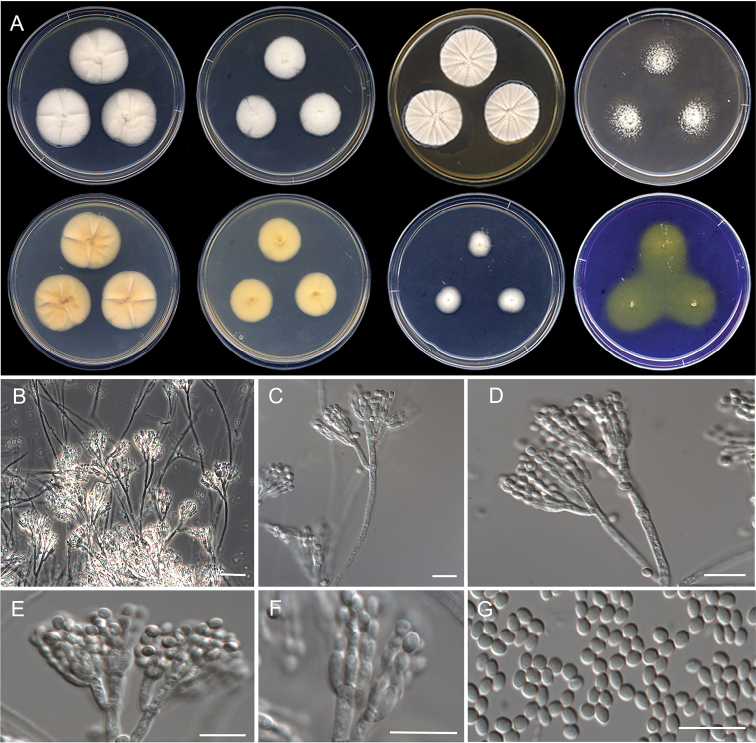
Morphological characters of *Penicilliumsubmersum* sp. nov. (ex-type FMR 17140). **A** colonies from left to right (top row) CYA, MEA, YES, and OA; (bottom row) CYA reverse, MEA reverse, DG18, and CREA**B–F** conidiophores on MEA**G** conidia. Scale Bars: 25 μm (**B**), 10 μm (**C–G**).

#### Subgeneric classification.

Subgenus Penicillium, sectionRobsamsonia, series *Urticicola*.

#### Description.

***Mycelium*** superficial and immersed composed of septate, smooth-walled, hyaline hyphae, 2–2.5 μm wide. ***Conidiophores*** mostly terverticillate, in minor proportion biverticillate and quarterverticillate; ***stipes*** smooth-walled, 29–142 × 1.5–2.5 μm; ***metulae*** divergent, mostly 2, occasionally 3 per stipe/branch, 5.5–7.5 × 1.5–4 μm; ***phialides*** 2–5 per metula, ampulliform, 4–5.5 × 1.5–2.5 μm; ***conidia*** smooth-walled, ellipsoidal, 3–3.5 × 2–2.5 μm.

#### Culture characteristics (7 d at 25 °C).

Colonies on CYA, 34–37 mm diam., elevated, with some radially furrow, floccose, light yellow (4A5) and yellowish white (4A2) towards periphery, margins entire, sporulation sparse, conidial masses grayish green (28C4); reverse golden brown (5D7) and orange (5A6) at periphery; soluble pigment absent. On MEA, 28–29 mm diam., slightly elevated, floccose, white (1A1) to light yellow (4A5) at periphery, margins entire, sporulation sparse, conidial masses grayish green (27C3); reverse light yellow (4A5); soluble pigment absent. On YES, 33–36 mm diam., slightly elevated at center, radially sulcate, velvety, light brown (6D4) and white (1A1) towards periphery, margins slightly lobate, sporulation sparse, conidial masses grayish green (28C3); reverse grayish orange (5B5); soluble pigment absent. On OA, 18–20 mm diam., elevated at center, fasciculate, yellowish white (4A2) and pale gray towards periphery, margins low and entire, sporulation abundant, conidial masses grayish green (28B3); reverse grayish yellow (4C5); soluble pigment absent. On DG18, 11–13 mm diam., elevated, floccose, white (1A1) at center, pale yellow (4A3) and grayish yellow (4C3) towards periphery, margins entire, sporulation abundant, conidial masses grayish green (27C3); reverse light yellow (4A5) and yellowish white (2A2) at periphery; soluble pigment absent. On CREA, 15–19 mm diam., flattened, floccose, white (1A1) and pale yellow (3A3), margins low and irregular, sporulation sparse, conidial masses grayish green (28B3–C3); reverse white (1A1) and pale yellow (3A3); soluble pigment absent, acid production strong.

#### Colony diameter on CYA after 7d (mm).

5 °C no growth, 15 °C 20–21, 20 °C 25–26, 30 °C 28–30, 35 °C 17–16, 37 °C 9–11, 40 °C no growth.

#### Distribution.

Spain.

#### Notes.

Species in sectionRobsamsonia were characterized by restricted to moderately fast growth rate on CYA at 25 °C (15–32 mm diam in 7 d) and lack or slow of growth on CYA at 30 °C (up to 19 mm diam) (Houbraken et al. 2016; Houbraken et al. 2020). However, the novel species showed faster growth rates on CYA at both temperatures (i.e., 34–37 mm and 28–30 mm diam., respectively). *Penicilliumsubmersum* shares morphological features with the other two species (*P.griseofulvum* and *P.dipodomycola*) of the series *Urticicola* where it is classified (Fig. 2), which consisted in having bi-, ter, or quarterverticillate, divergent, smooth-walled conidiophores and short phialides (up to 7 µm) (Houbraken et al. 2020). However, *P.submersum* shows the shortest phialides within the group (4–5.5 vs. 5–7 µm). In addition, our species has strong acid production on CREA, in contrast to the lack of acid production of *P.griseofulvum* and *P.dipodomycola* in the same medium (Houbraken et al. 2016, 2020); and colony reverse on CYA and YES in *P.submersum* is golden brown to orange and grayish orange, respectively, while in *P.griseofulvum* and *P.dipodomycola* it is beige brown to dark brown in both culture media (Houbraken et al. 2020). Furthermore, *P.griseofulvum* differs from *P.submersum* in its gray colony color, especially on CYA, which is in shades of yellow in our species.

### ﻿ Penicillium tardochrysogenum

Taxon classificationFungiEurotialesAspergillaceae

Frisvad, Houbraken & Samson . Persoonia 29: 93. 2012.

Figure 15

#### Subgeneric classification.

Subgenus Penicillium, sectionChrysogena, series *Chrysogena*.

#### Description.

***Mycelium*** superficial and immersed composed of septate, smooth-walled, hyaline hyphae, 2.5–5.5 μm wide. ***Conidiophores*** biverticillate, terverticillate or quaterverticillate; ***stipes*** smooth-walled, 40–200 × 2.5–4 μm; ***metulae*** appressed to slightly divergent, 2–4 per branch or stipe, vesiculate, 6–12.5 × 2–4 μm (vesicle up to 4.5 μm wide); ***phialides*** 3–6 per metulae, ampulliform, 5.5–7.5 × 1.5–2.5 μm; ***conidia*** smooth-walled, globose to subglobose, 2.5–3 × 2.5–3 μm.

**Figure 15.**
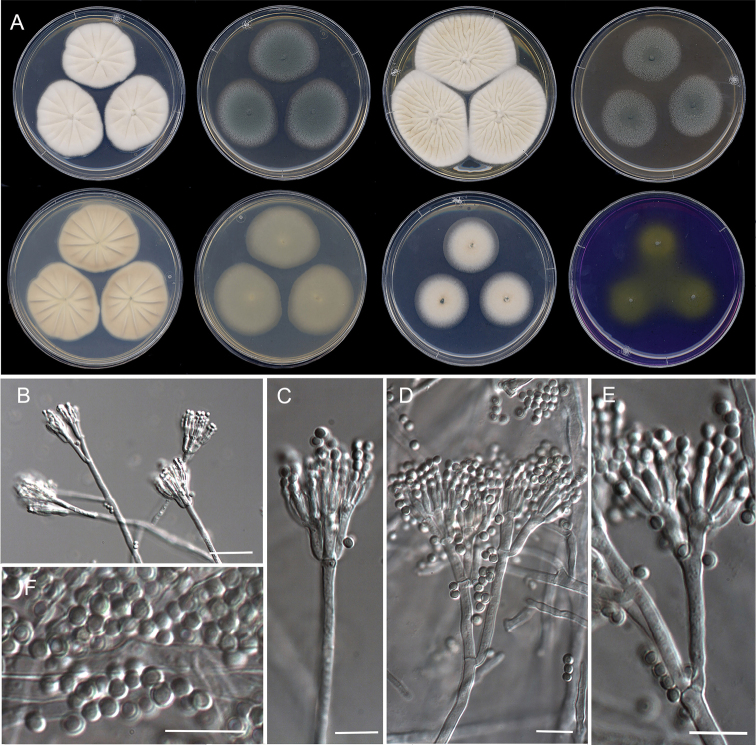
Morphological characters of *Penicilliumtardochrysogenum* (FMR 17137). **A** colonies from left to right (top row) CYA, MEA, YES, and OA; (bottom row) CYA reverse, MEA reverse, DG18, and CREA**B–E** conidiophores on MEA**F** conidia. Scale Bars: 25 μm (**B**), 10 μm (**C–F**).

#### Culture characteristics (7 d at 25 °C).

Colonies on CYA reaching 41–43 mm diam., slightly raised at center, floccose, radially sulcate, yellowish white (1A2) at center to orange white (6A2) and white (1A1) towards periphery, margins slightly lobate, sporulation sparse, conidial masses grayish green (28B3); reverse champagne (4B4) at center to red-haired (6C4) towards periphery; soluble pigment absent. On MEA, 38–39 mm diam., flattened, velvety, grayish green (26D2-E4), white (1A1) towards periphery, margins low and slightly fimbriate, sporulation abundant, conidial masses dull green (25D4); reverse colorless; soluble pigment absent. On YES, 56–57 mm diam., slightly raised at center, floccose, radially sulcate, yellowish white (4A2) at center to champagne (4B4) towards periphery, margins entire, sporulation sparse, conidial masses grayish green (27B3); reverse amber yellow (4B6) at center to maize (yellow) (4A5) towards periphery; soluble pigment absent. On OA, 35–37 mm diam., flattened, velvety, dark green (28F8) at center to greenish gray (28D4) and white (1A1) towards periphery, margins low and entire, sporulation abundant, conidial masses dull green (25D3); reverse honey yellow (4D6) at center and sand yellow (4B3) towards periphery; soluble pigment absent. On DG18, 30–32 mm diam., flattened, floccose, dark green (27F8) at center to pale orange (5A3) and white (1A1) towards periphery, margins low and entire, sporulation abundant, conidial masses grayish green (28C3) at the center; reverse wax yellow (3B5) at center to white (1A1) towards periphery; soluble pigment absent. On CREA, 24–25 mm diam., flattened, floccose, jade green (27E5) at center to yellowish green (30B8) towards periphery, margins slightly fimbriate, sporulation sparse, conidial masses grayish green (27C3–C4); reverse yellowish white (30B8), soluble pigment absent and acid production moderately strong.

#### Colony diameter on CYA after 7d (mm).

5 °C 3–4, 15 °C 23–24, 20 °C 27–28, 30 °C 31–33, 35 °C 16–19, 37 °C 8–9, 40 °C no growth.

#### Specimen examined.

Spain, Catalonia, Montsant Natural Park, Siurana’s Swamp, from sediments, Feb 2018, *E. Carvalho* & *J. Gené* (FMR 17137).

#### Distribution.

Antarctica and Spain.

#### Notes.

Although *P.tardochrysogenum* was introduced based only on the type specimen collected in the Antarctica, the species was later described as endemic of that continent since it was isolated at high densities on rocks from several Islands and Continental Antarctica (Houbraken et al. 2012; Alves et al. 2019). The Spanish isolate from freshwater sediments represents the first report of this species in temperate regions. Of note, however, is that recently the species has also been reported from historical manuscripts preserved in Iraq (Jasim et al. 2019), but only the ITS barcode was used for confirming the identity of isolates, a well-known gene marker unable to distinguish between closely related penicillia (Houbraken et al. 2012; Visagie et al 2014a). *Penicilliumtardochrysogenum* belongs to series *Chrysogena* and is closely related to *P.allii-sativi*, *P.chrysogenum*, *P.rubens* and *P.vanluykii* (Fig. 1), but it was distinguished from these species and other members of the series by more restricted and floccose colonies on MEA, the lack of sporulation on YES and the production of finely roughened conidia (Houbraken et al. 2012). Despite the high sequence similarity of the markers analyzed with the ex-type strain of *P.tardochrysogenum*, our isolate showed some phenotypic variation regarding the protologue; i.e., faster growth rate after 7 d on MEA (38–39 vs. 18–24 mm), sporulation (sparse) on YES, smooth-walled conidia, and shorter stipes (40–200 × 2.5–4 vs. 150–400 × 2–3 µm) and metulae (6–12.5 × 2–4 vs. 10–13(–18) × 2.5–3.5 µm). These differences suggest that more specimens should be examined for a more accurate morphological characterization of this fungus.

### ﻿ Penicillium vaccaeorum

Taxon classificationFungiEurotialesAspergillaceae

Quintanilla, Mycopathol. 80: 74. 1982.

109999

Figure 16

=Penicilliumlacussarmientei Ramírez, Mycopathol. 96: 29. 1986.

#### Type.

Spain, Valladolid, San Miguel del Arroyo, from sandy soil under pine tree; J.A. Quintanilla (***holotype***CBS H-148.83, cultures ex-type CBS 148.83, DTO 9E2, CECT 2753).

**Figure 16.**
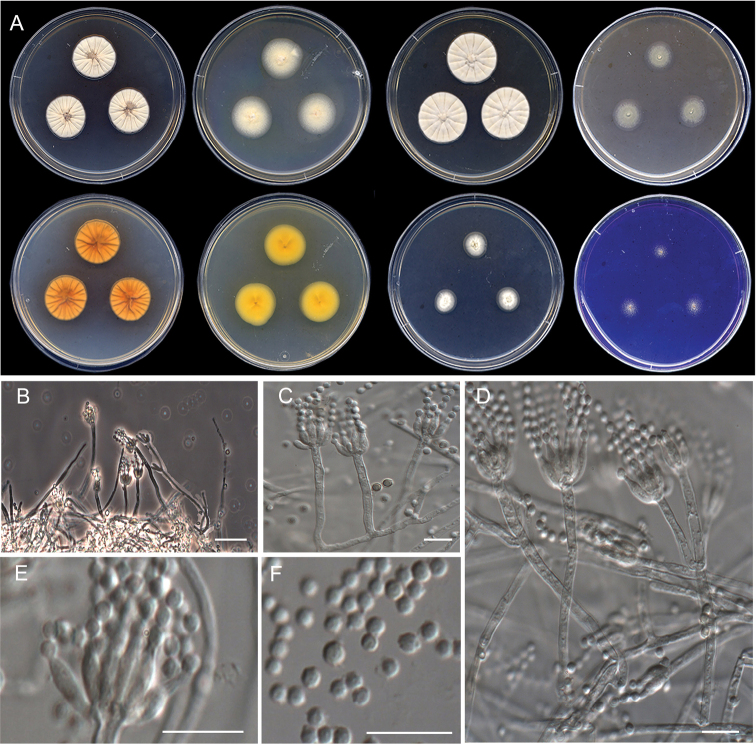
Morphological characters of *Penicilliumvaccaeorum* (FMR 17967). **A** colonies from left to right (top row) CYA, MEA, YES, and OA; (bottom row) CYA reverse, MEA reverse, DG18, and CREA**B–E** conidiophores on MEA**F** conidia. Scale Bars: 25 μm (**B**), 10 μm (**C–F**).

#### Subgeneric classification.

Subgenus Aspergilloides, sectionCitrina, series *Roseopurpurea*

#### Description.

***Mycelium*** superficial and immersed composed of septate, smooth-walled, hyaline hyphae of 1.5–2.5 μm wide. ***Conidiophores*** monoverticillate, rarely biverticillate and divaricate; ***stipes*** smooth-walled, vesiculate, 22.5–103 × 1.5–2.5 μm (vesicle up to 4.5 µm); ***metulae*** divergent 2–3, unequal in length, 7–37 × 1.5–3 µm; ***phialides*** 2–5 per stipe/metula, ampulliform, 6–8.5 × 2–2.5 μm; ***conidia*** smooth- or finely roughened, globose, 2–2.5 × 2–2.5 μm.

#### Culture characteristics (7 d at 25 °C).

Colonies on CYA, 20–22 mm diam., slightly raised, velvety, radially sulcate, dull red (8C3) at center to light yellow (4A5) and white (1A1) towards periphery, margins slightly undulate, sporulation sparse, conidial masses grayish green (28B3); reverse brownish orange (5C6); with reddish soluble pigment. On MEA, 24–27 mm diam., slightly elevated, velvety, light yellow (4A5) and pale orange (5A2) at periphery, margins low and entire, sporulation sparse, conidial masses grayish green (27C4); reverse golden yellow (5B7) and reddish-yellow (4A6) at periphery; soluble pigment absent. On YES, 30–32 mm diam., slightly raised at center, velvety, radially sulcate, pale yellow (3A3) to white (1A1) and brownish orange (5C3) towards periphery, margins slightly undulate, sporulation abundant, conidial masses grayish green (28B3); reverse brownish yellow (5C8); soluble pigment absent. On OA, 18–20 mm diam., flattened, velvety, dark green (28F4) to light gray (25D1) and white (1A1) towards periphery, margins low and entire, sporulation abundant, conidial masses dull green (25D3); reverse brown (6E4) and yellowish gray (4B2) at periphery; soluble pigment absent. On DG18, 12–13 mm, slightly raised at center, velvety, radially sulcate, white (1A1) and yellowish white (1A2) towards periphery, margins regular, sporulation sparse, conidial masses grayish green (27C3); reverse light yellow (4A5) and white (1A1) at periphery; soluble pigment absent. On CREA, 9–11 mm diam., flattened, floccose, yellowish green (29B7) and white (1A1) towards periphery, margins entire, sporulation sparse, conidial masses grayish green (28B3); reverse dark gray (1F1); soluble pigment and production of acid absent.

#### Colony diameter on CYA after 7d (mm).

On CYA: 5 °C no growth, 15 °C 13–14, 20 °C 16–17, 30 °C 18–20, 35 °C 6–11, 37 °C no growth, 40 °C no growth.

#### Specimens examined.

Spain, Catalonia, Fogars de Montclús, La Costa de l’Infern, from stream sediments, Oct 2018, *D. Torres* (FMR 17531); Fogars de Montclús, La Costa de l’Infern, from stream sediments, Oct 2018, *D. Torres* (FMR 17534); Aitona, Segre River, from sediments, Dec 2020, *D. Torres* & *J. Gené* (FMR 18100); La Granja d’Escarp, Segre River, from sediments, Dec 2020, *D. Torres* & *J. Gené* (FMR 18123); Balearic Islands, Mallorca, Serra de Tramontana, from stream sediments, Dec 2018, *J. F. Cano* (FMR 17616); Basque Country, from stream sediments, Aug 2019, *J. Gené* (FMR 17967).

#### Distribution.

Argentina, Canada, Chile, Spain, The Netherlands and Turkey.

#### Notes.

*Penicilliumvaccaeorum* and *P.lacussarmientei*, two species described from sandy soils in Spain and Chile (Quintanilla 1982; Ramírez 1986), respectively, were considered synonyms of *P.roseopurpureum* by Frisvad et al. (1990), noting that both species were fast growing variants of *P.roseopurpureum*. Later on, based on that criterion and the lack of morphological differences, Houbraken et al. (2011) considered the two former species synonyms of *P.sanguifluum* despite some sequence variation where *P.vaccaeorum* and *P.lacussarmientei* clustered together in a clade sister to that of *P.sanguifluum*. Our phylogeny correlates with Houbraken et al. (2011) who found the same topology. Having the opportunity to examine specimens from both monophyletic sister clades (Fig. 8), we observed consistent phenotypic features to distinguish them. For instance, isolates of *P.vaccaeorum* had longer stipes (up to 103 μm; up to 120 μm in the protologue of the species) (Quintanilla 1982), they were able to grow on CYA at 35 °C (6–11 mm diam. after 7 d), had good sporulation and faster growth on YES agar (30–32 mm diam. 7 d) and more restricted on DG18 (12–13 mm diam. 7 d). In contrast, isolates of the *P.sanguifluum* clade showed considerably shorter conidiophores (15–50 µm long), they were unable to grow above 30 °C, and the colonies on YES and DG18 showed sparsely or absent sporulation and attained 18–28 mm and 16–22 mm diam., respectively (Houbraken et al. 2011). Hence, genetic and phenotypic differences support the reinstatement of *P.vaccaeorum* as an accepted species, with *P.lacussarmientei* considered synonym. This species together with *P.sanguifluum* and *P.roseopurpureum* are classified in series *Roseopurpurea*, which differs from almost all series of the sectionCitrina by species’ monoverticillate conidiophores. The only other series in the section with monoverticillate conidiophores is *Gallaica*, represented exclusively by *P.gallaicum*, which differs from the former series mainly by the production of sclerotia (Houbraken et al. 2020).

According to the revised data, *P.vaccaeorum* occurs worldwide, and is commonly isolated from sandy soils of beaches and forests, and even associated with ants (Table 1).

## ﻿Discussion

Due to nomenclatural revisions of *Penicillium*, and efforts to release extensive reference sequences for both ex-types and other reference strains (Houbraken and Samson 2011; Visagie et al. 2014a; Houbraken et al. 2020), the number of *Penicillium* species newly described in the last decade has significantly increased, particularly with the examination of fungi recovered from poorly studied substrates or unexplored areas (sensu Hawksworth and Lücking 2017; Wijayawardene et al. 2020). Although *Penicillium* species are commonly isolated from aquatic environments (Niewolak 1975; Tóthová 1999; Amaral-Zettler et al. 2002), studies exploring their diversity are scarce. For instance, Heo et al. (2019) recovered *P.brasilianum*, *P.crustosum*, *P.expansum*, *P.oxalicum* and *P.piscarium* from freshwater environments in Korea. Only recently, *Penicillium* diversity has begun to be investigated in marine sediments (Gonçalves et al. 2013; Kirichuk et al. 2017; Ogaki et al. 2020a), and some novel species have even been described from this substrate, such as *P.piltunense*, *P.ochotense* and *P.attenuatum* (Kirichuk et al. 2017), although they are currently considered conspecific with *P.antarcticum* (Visagie et al. 2021).

Several studies reveal that the fungal biomass and, subsequently, ascomycetes inhabiting freshwater sediments are fundamental to the decomposition of deposited organically bound C and N, using carbohydrates, phenolic compounds and carboxylic acids as carbon sources (Huang et al. 2010; Zhang et al. 2013; Zhang et al. 2014). The penicillia might also contribute greatly to this activity, although further studies are needed to define the spectrum of species and their role in this particular substrate.

In our contribution to the diversity of *Penicillium* from freshwater sediments, we identified several interesting species, such as *P.heteromorphum* and *P.tardochrysogenum* only so far known from China and Antarctica, respectively (Kong and Qi 1988; Alves et al. 2019). Our finding reveals a wider distribution of these taxa, being first reports in freshwater sediment samples, as in the case of the resurrected species *P.vaccaeorum* and its counterpart *P.sanguifluum*. These two latter species (classified in sectionCitrina) seem to be common penicillia inhabiting soil (Houbraken et al. 2011). Furthermore, we recovered several isolates representative of new species described above as *P.ausonanum*, *P.guarroi*, *P.irregulare*, *P.sicoris* and *P.submersum*. Of note is that of the fifteen *Penicillium* isolates investigated (Table 1), twelve were obtained from PDA supplemented with 0.2% cycloheximide, a culture technique previously used to recover keratinophylic or extremotolerant fungi such as black yeasts from various substrates (Ulfig et al. 1997; Salgado et al. 2004; Madrid et al. 2016). Isolates obtained using this culture medium, and therefore resistant to a relatively high concentration of cycloheximide, corresponded to *P.ausonanum*, *P.irregulare*, *P.submersum*, *P.tardochrysogenum*, as well as all those identified as *P.vaccaeorum* and *P.sanguifluum*. Cycloheximide tolerance in *Penicillium* has been previously studied by Seifert and Giuseppin (2000), even recently two novel species, *P.krskae* and *P.silybi*, have been described as resistant to cycloheximide (Labuda et al. 2021). Other fungi with the ability to grow at high doses of that antimicrobial agent can cause human infections, such as numerous dermatophytes, black yeasts, or members in the *Ophiostomatales* (de Hoog et al. 2020). However, the meaning of the cycloheximide resistance in penicillia would deserve further studies, for instance at genomic level. On the other hand, the isolates of *P.guarroi*, *P.heteromorphum* and *P.sicoris* were obtained from DRBC. It is well-known that this culture medium restricts fast-growing moulds due mainly to the Rose Bengal effect, allowing them to recover fungi with slower growth rates, a feature shown by the mentioned species in comparison with other *Penicillium* species that have been previously recovered from aquatic environments (Gonçalves et al. 2019; Heo et al. 2019). The efficacy of the DRBC for the detection of filamentous fungi and yeasts in aquatic environments was proven in a study investigating the yield across different media, in which it was found to be the best in quantity and diversity of fungi detection in different water samples tested (Pereira et al. 2010). According to our experience, it is important to use different media in order to investigate and recover the greatest diversity of species as possible from environmental samples.

Most of the new species described here were mainly recovered from samples collected in rivers and streams of the north-western Mediterranean region, an area recognized as one of the most species-rich regions in Southern Europe (Máiz-Tomé et al. 2017; Rundel et al. 2018). This fact was particularly highlighted in the study of Guevara-Suárez et al. 2020 on species diversity of coprophilous penicillium-like fungi, from which the highest number of species identified and novel taxa described were recovered from samples collected in Mediterranean localities. Therefore, regarding fluvial sediments as an accumulative substrate in biodiversity of the environment, it is not surprising that freshwater sediments from the Mediterranean region comprise a great reservoir of novel fungal lineages in general.

## Supplementary Material

XML Treatment for Penicillium ausonanum

XML Treatment for Penicillium guarroi

XML Treatment for Penicillium heteromorphum

XML Treatment for Penicillium irregulare

XML Treatment for Penicillium sicoris

XML Treatment for Penicillium submersum

XML Treatment for Penicillium tardochrysogenum

XML Treatment for Penicillium vaccaeorum

## Supplementary materials

Supplementary material 1 Figures S1–S9 Data type phylogenetic trees Explanation note Additional phylogenetic trees of the *Penicillium* series corresponding to the individual analyses of the different molecular markers with additional reference strains representing the species closely related to penicillia described here. https://binary.pensoft.net/file/640616This dataset is made available under the Open Database License (http://opendatacommons.org/licenses/odbl/1.0/). The Open Database License (ODbL) is a license agreement intended to allow users to freely share, modify, and use this Dataset while maintaining this same freedom for others, provided that the original source and author(s) are credited.D Torres-Garcia, J Gené, D García
