# Supplementary material for: ﻿New and interesting species of Penicillium (Eurotiomycetes, Aspergillaceae) in freshwater sediments from Spain
Source: MycoKeys. 2022 Feb 1;86:103–45. doi: 10.3897/mycokeys.86.73861 (PMC8825427; doi:10.3897/mycokeys.86.73861)
Supplement: Supplementary material 1 — Figures S1–S9 [file mycokeys-86-103-s001.pdf]

SUPPLEMENTARY MATERIAL-Figures S1-S9

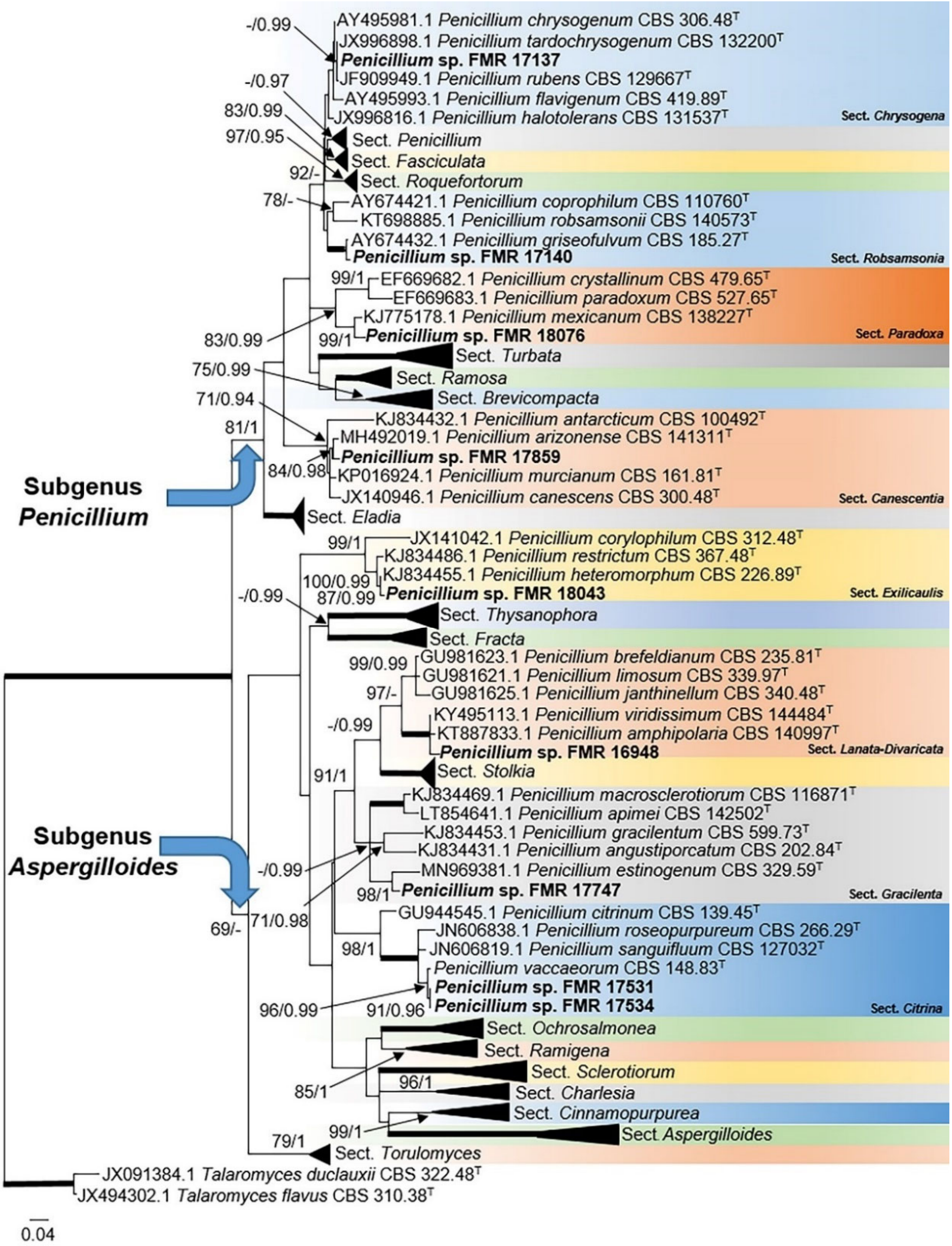

Figure S1. ML tree of all *Penicillium* sections inferred from *tub*2 including the strains recovered from freshwater sediments studied in this work. Determined by MEGA software, the best nucleotide substitution model for ML analysis was K2+G+I. Determined by jModelTest, the best nucleotide substitution model for BI analysis was HKY+G+I. The aligned dataset was 525 bp long, with 375 variable sites and 357 phylogenetically informative. Branch lengths are proportional to phylogenetic distance. Some of the larger branches were condensed, with the proportions showed above the parallel diagonal lines. Bold branches indicate bs/pp values 100/1. Bootstrap support values/Bayesian posterior probability scores above 69 %/0.94 are indicated on the nodes. The tree is rooted to *Talaromyces flavus* CBS 310.38 and *Talaromyces duclauxii* CBS 322.48. ^{T}= Ex-type strain.

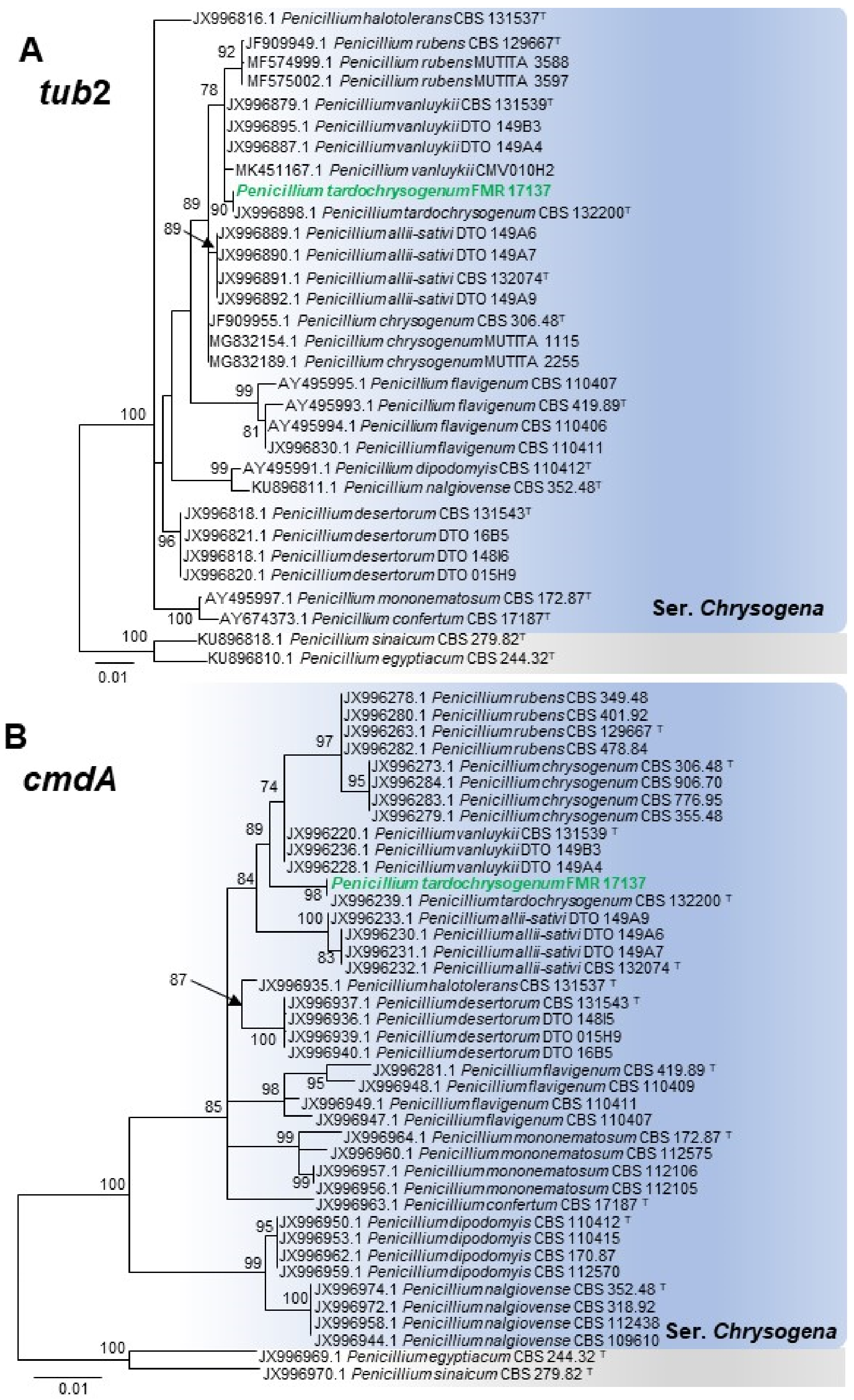

Figure S2: RAxML tree of selected strains of *Penicillium* in section *Chrysogena*, series *Chrysogena,* inferred from (**A**) *tub*2 and (**B**) *cmdA*. Determined by Mega software 6.0., K2+G was the best substitution model for both phylogenetic markers. The aligned dataset was 560 and 496 bp long for *tub*2 and *cmdA*, respectively, with 55 variable sites and 35 phylogenetically informative sites for *tub*2, and 83 variable sites and 58 phylogenetically informative sites for *cmdA*. Branch lengths are proportional to phylogenetic distance. Bootstrap support values above 70% are indicated on the nodes. The trees are rooted to *P. egyptiacum* CBS 244.32 and *P. sinaicum* CBS 279.82. ^{T} = Ex-type strain.

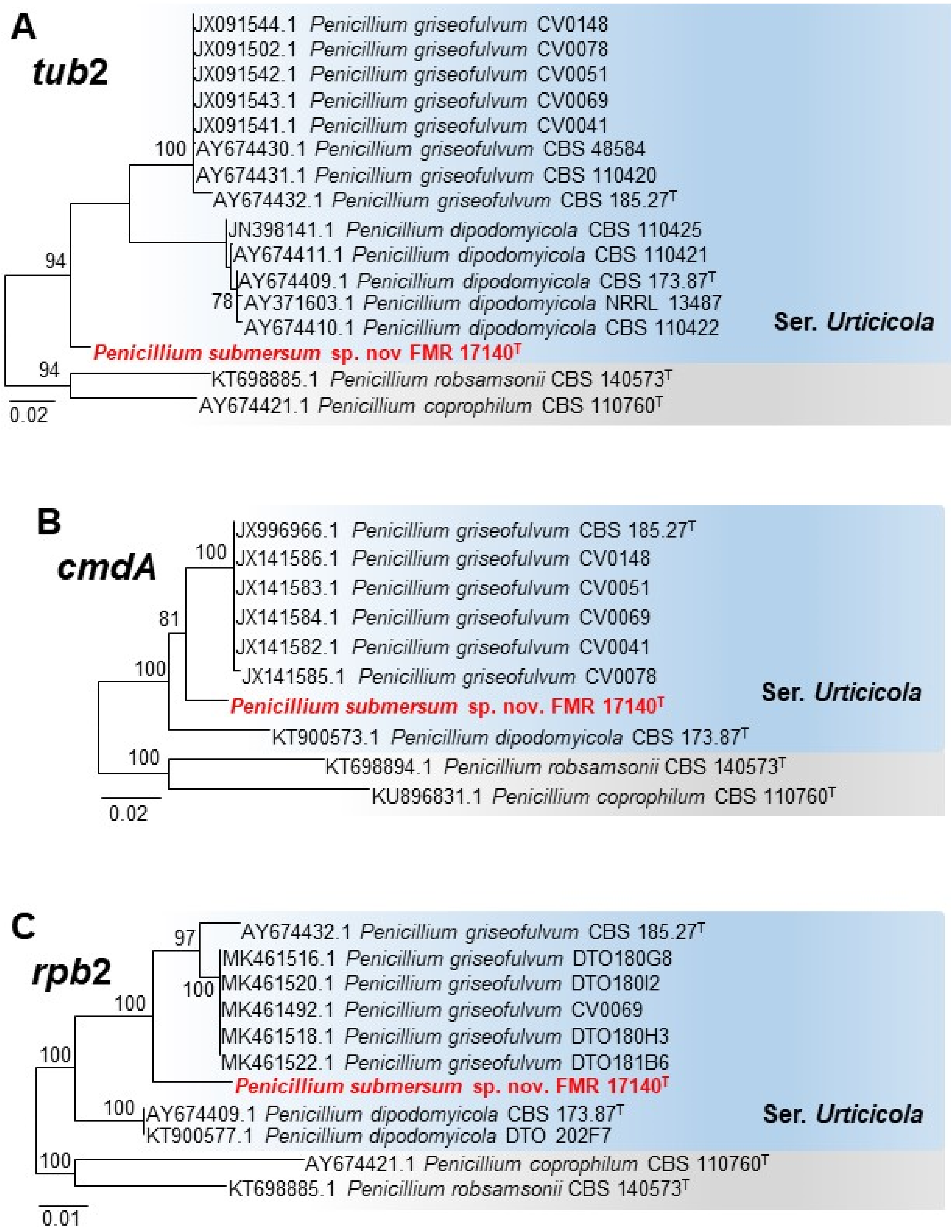

Figure S3: RAxML tree of selected strains of *Penicillium* in section *Robsamsonia*, series *Urticicola*, inferred from (**A**) *tub*2, (**B**) *cmdA* and (**C**) *rpb*2. Determined by Mega software 6.0., K2+G was the best substitution model for all phylogenetic markers. The aligned dataset was 462, 503 and 936 bp long for *tub*2, *cmdA* and *rpb*2, respectively, with 75 variable sites and 43 phylogenetically informative sites for *tub*2, 85 variable sites and 29 phylogenetically informative sites for *cmdA*, and 105 variable sites and 48 phylogenetically informative sites for *rpb*2. Branch lengths are proportional to phylogenetic distance. Bootstrap support values above 70% are indicated on the nodes. The trees are rooted to *P. coprophilum* CBS 110760 and *P. robsamsonii* CBS 140573. ^{T} = Ex-type strain.

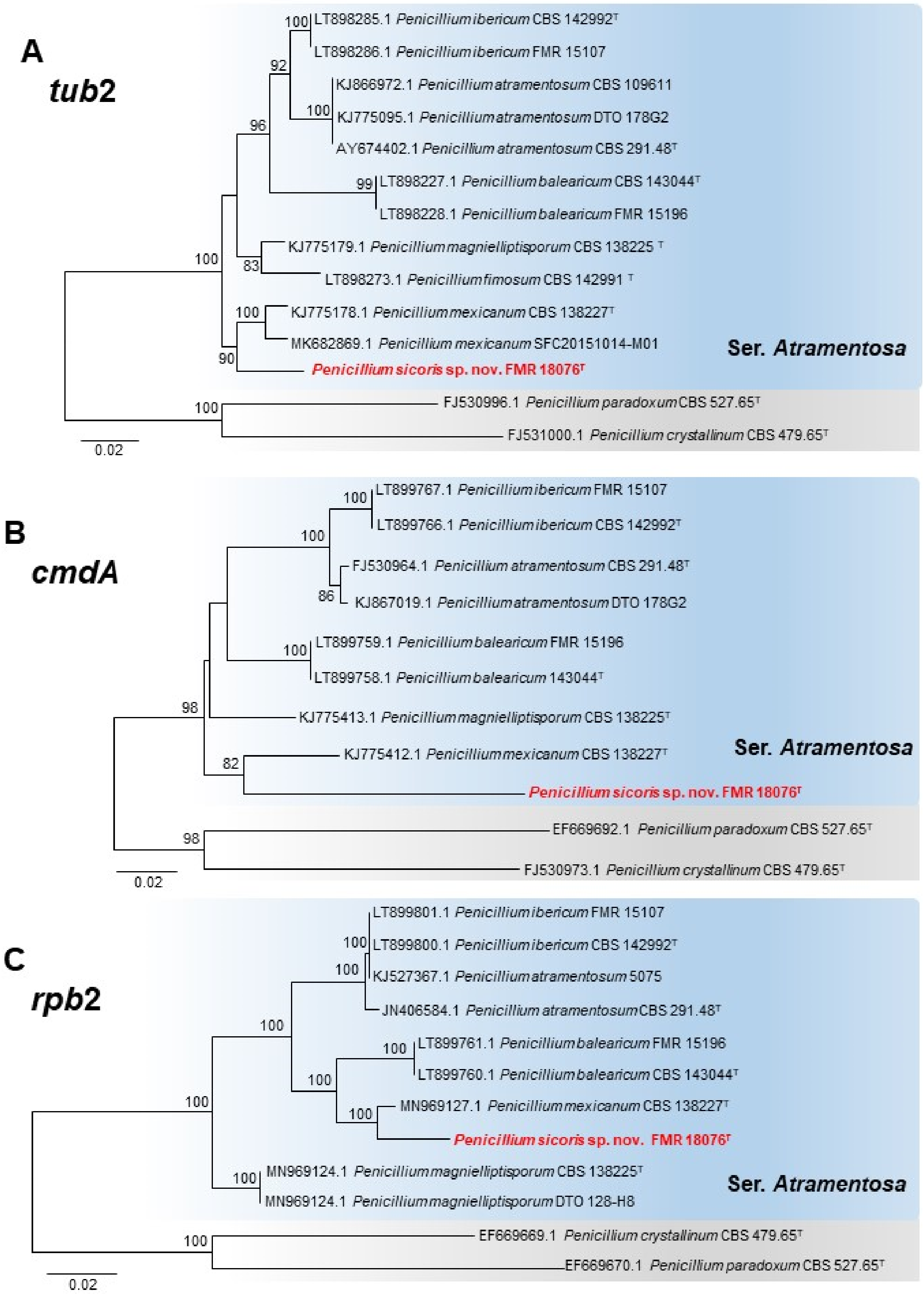

Figure S4: RAxML tree of selected strains of *Penicillium* in section *Paradoxa*, series *Atramentosa*, inferred from (**A**) *tub*2, (**B**) *cmdA* and (**C**) *rpb*2. Determined by Mega software 6.0., K2+G was the best substitution model for all phylogenetic markers. The aligned dataset was 491, 525 and 929 bp long for *tub*2, *cmdA* and *rpb*2, respectively, with 120 variable sites and 62 phylogenetically informative sites for *tub*2, 152 variable sites and 62 phylogenetically informative sites for *cmdA*, and 183 variable sites and 103 phylogenetically informative sites for *rpb*2. Branch lengths are proportional to phylogenetic distance. Bootstrap support values above 70% are indicated on the nodes. The trees are rooted to *P. crystallinum* CBS 479.65 and *P. paradoxum* CBS 527.65. ^{T} = Ex-type strain.

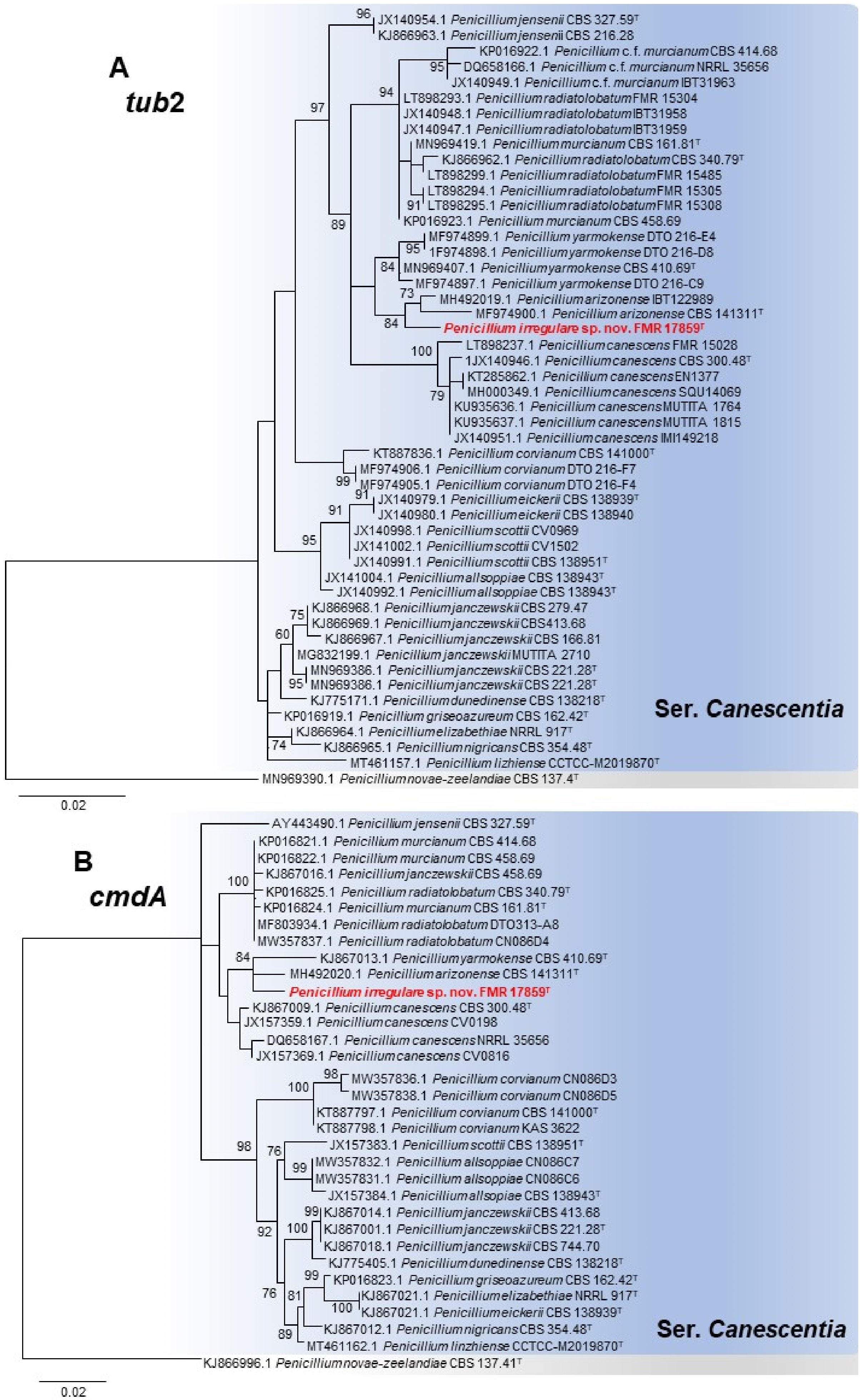
A
tub2
JX140954.1 Penicillium jensenii CBS 327.59T
KJ866963.1 Penicillium jensenii CBS 216.28
KP016922.1 Penicillium c.f. murcianum CBS 414.68
DQ658166.1 Penicillium c.f. murcianum NRRL 35656
JX140949.1 Penicillium c.f. murcianum IBT31963
LT898293.1 Penicillium radiatolobatum FMR 15304
JX140948.1 Penicillium radiatolobatum IBT31958
JX140947.1 Penicillium radiatolobatum IBT31959
MN969419.1 Penicillium murcianum CBS 161.81T
KJ866962.1 Penicillium radiatolobatum CBS 340.79T
LT898299.1 Penicillium radiatolobatum FMR 15485
LT898294.1 Penicillium radiatolobatum FMR 15305
LT898295.1 Penicillium radiatolobatum FMR 15308
KP016923.1 Penicillium murcianum CBS 458.69
MF974899.1 Penicillium yarmokense DTO 216-E4
1F974898.1 Penicillium yarmokense DTO 216-D8
MN969407.1 Penicillium yarmokense CBS 410.69T
MF974897.1 Penicillium yarmokense DTO 216-C9
MH492019.1 Penicillium arizonense IBT122989
MF974900.1 Penicillium arizonense CBS 141311T
Penicillium irregulare sp. nov. FMR 17859T
LT898237.1 Penicillium canescens FMR 15028
1JX140946.1 Penicillium canescens CBS 300.48T
KT285862.1 Penicillium canescens EN1377
MH000349.1 Penicillium canescens SQU14069
KU935636.1 Penicillium canescens MUTITA 1764
KU935637.1 Penicillium canescens MUTITA 1815
JX140951.1 Penicillium canescens IMI149218
KT887836.1 Penicillium corvianum CBS 141000T
MF974906.1 Penicillium corvianum DTO 216-F7
MF974905.1 Penicillium corvianum DTO 216-F4
JX140979.1 Penicillium eickerii CBS 138939T
JX140980.1 Penicillium eickerii CBS 138940
JX140998.1 Penicillium scottii CV0969
JX141002.1 Penicillium scottii CV1502
JX140991.1 Penicillium scottii CBS 138951T
JX141004.1 Penicillium allsoppiae CBS 138943T
JX140992.1 Penicillium allsoppiae CBS 138943T
KJ866968.1 Penicillium janczewskii CBS 279.47
KJ866969.1 Penicillium janczewskii CBS413.68
KJ866967.1 Penicillium janczewskii CBS 166.81
MG832199.1 Penicillium janczewskii MUTITA 2710
MN969386.1 Penicillium janczewskii CBS 221.28T
MN969386.1 Penicillium janczewskii CBS 221.28T
KJ775171.1 Penicillium dunedinense CBS 138218T
KP016919.1 Penicillium griseoazureum CBS 162.42T
KJ866964.1 Penicillium elizabethiae NRRL 917T
KJ866965.1 Penicillium nigricans CBS 354.48T
MT461157.1 Penicillium lizhiense CCTCC-M2019870T
MN969390.1 Penicillium novae-zeelandiae CBS 137.4T
Ser. Canescentia
96
95
94
97
89
91
95
84
73
84
100
79
99
91
91
95
75
60
95
74
0.02
B
cmdA
AY443490.1 Penicillium jensenii CBS 327.59T
KP016821.1 Penicillium murcianum CBS 414.68
KP016822.1 Penicillium murcianum CBS 458.69
KJ867016.1 Penicillium janczewskii CBS 458.69
KP016825.1 Penicillium radiatolobatum CBS 340.79T
KP016824.1 Penicillium murcianum CBS 161.81T
MF803934.1 Penicillium radiatolobatum DTO313-A8
MW357837.1 Penicillium radiatolobatum CN086D4
KJ867013.1 Penicillium yarmokense CBS 410.69T
MH492020.1 Penicillium arizonense CBS 141311T
Penicillium irregulare sp. nov. FMR 17859T
KJ867009.1 Penicillium canescens CBS 300.48T
JX157359.1 Penicillium canescens CV0198
DQ658167.1 Penicillium canescens NRRL 35656
JX157369.1 Penicillium canescens CV0816
MW357836.1 Penicillium corvianum CN086D3
MW357838.1 Penicillium corvianum CN086D5
KT887797.1 Penicillium corvianum CBS 141000T
KT887798.1 Penicillium corvianum KAS 3622
JX157383.1 Penicillium scottii CBS 138951T
MW357832.1 Penicillium allsoppiae CN086C7
MW357831.1 Penicillium allsoppiae CN086C6
JX157384.1 Penicillium allsopiae CBS 138943T
KJ867014.1 Penicillium janczewskii CBS 413.68
KJ867001.1 Penicillium janczewskii CBS 221.28T
KJ867018.1 Penicillium janczewskii CBS 744.70
KJ775405.1 Penicillium dunedinense CBS 138218T
KP016823.1 Penicillium griseoazureum CBS 162.42T
KJ867021.1 Penicillium elizabethiae NRRL 917T
KJ867021.1 Penicillium eickerii CBS 138939T
KJ867012.1 Penicillium nigricans CBS 354.48T
MT461162.1 Penicillium linzhiense CCTCC-M2019870T
KJ866996.1 Penicillium novae-zeelandiae CBS 137.41T
Ser. Canescentia
100
84
98
100
98
76
99
99
100
92
99
76
81
100
89
0.02

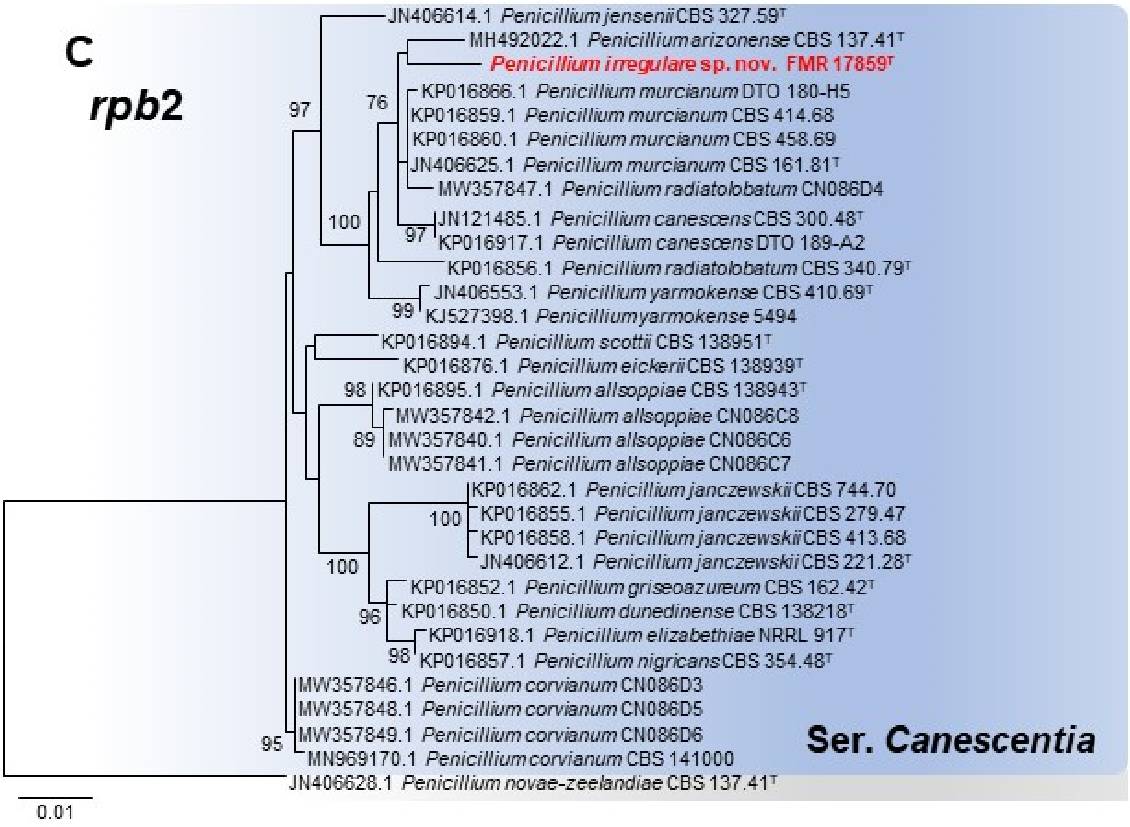

Figure S5: RAxML tree of selected strains of *Penicillium* in section *Canescentia*, series *Canescentia*, inferred from (**A**) *tub*2, (**B**) *cmdA* and (**C**) *rpb*2. Determined by Mega software 6.0., K2+G was the best substitution model for all phylogenetic markers. The aligned dataset was 459, 538 and 915 bp long for *tub*2, *cmdA* and *rpb*2, respectively, with 90 variable sites and 48 phylogenetically informative sites for *tub*2, 110 variable sites and 59 phylogenetically informative sites for *cmdA*, and 124 variable sites and 58 phylogenetically informative sites for *rpb*2. Branch lengths are proportional to phylogenetic distance. Bootstrap support values above 70% are indicated on the nodes. The trees are rooted to *P. novae-zeelandiae* CBS 137.41. T = Ex-type strain.

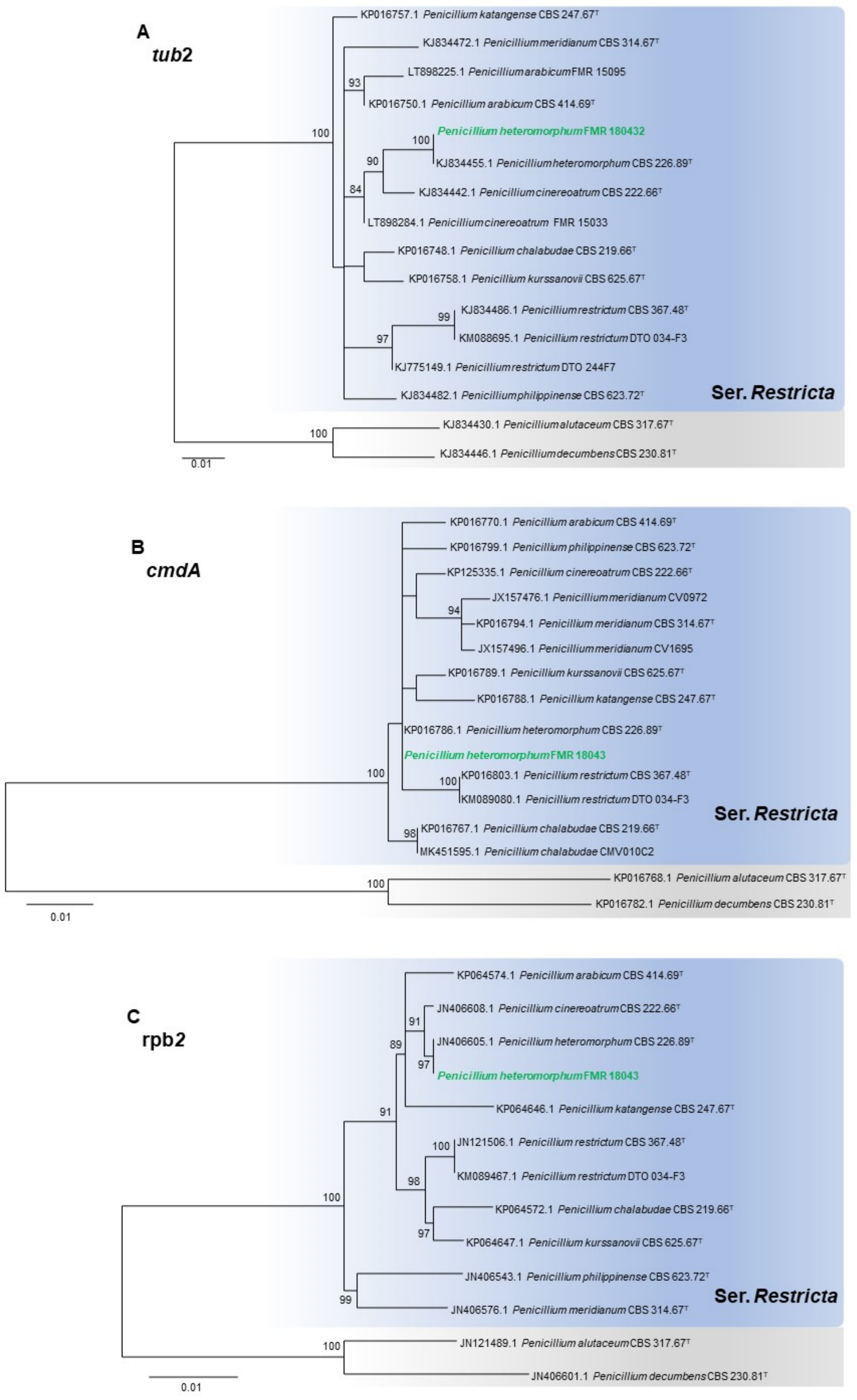

Figure S6: RAxML tree of selected strains of *Penicillium* in section *Exilicaulis*, series *Restricta*, inferred from (**A**) *tub*2, (**B**) *cmdA* and (**C**) *rpb*2. Determined by Mega software 6.0., K2+G was the best substitution model for all phylogenetic markers. The aligned dataset was 452, 508 and 934 bp long for *tub*2, *cmdA* and *rpb*2, respectively, with 75 variable sites and 47 phylogenetically informative sites for *tub*2, 77 variable sites and 45 phylogenetically informative sites for *cmdA*, and 106 variable sites and 51 phylogenetically informative sites for *rpb*2. Branch lengths are proportional to phylogenetic distance. Bootstrap support values above 70% are indicated on the nodes. The trees are rooted to *P. alutaceum* CBS 317.67 and *P. decumbens* CBS 230.81 ^{T} = Ex-type strain.

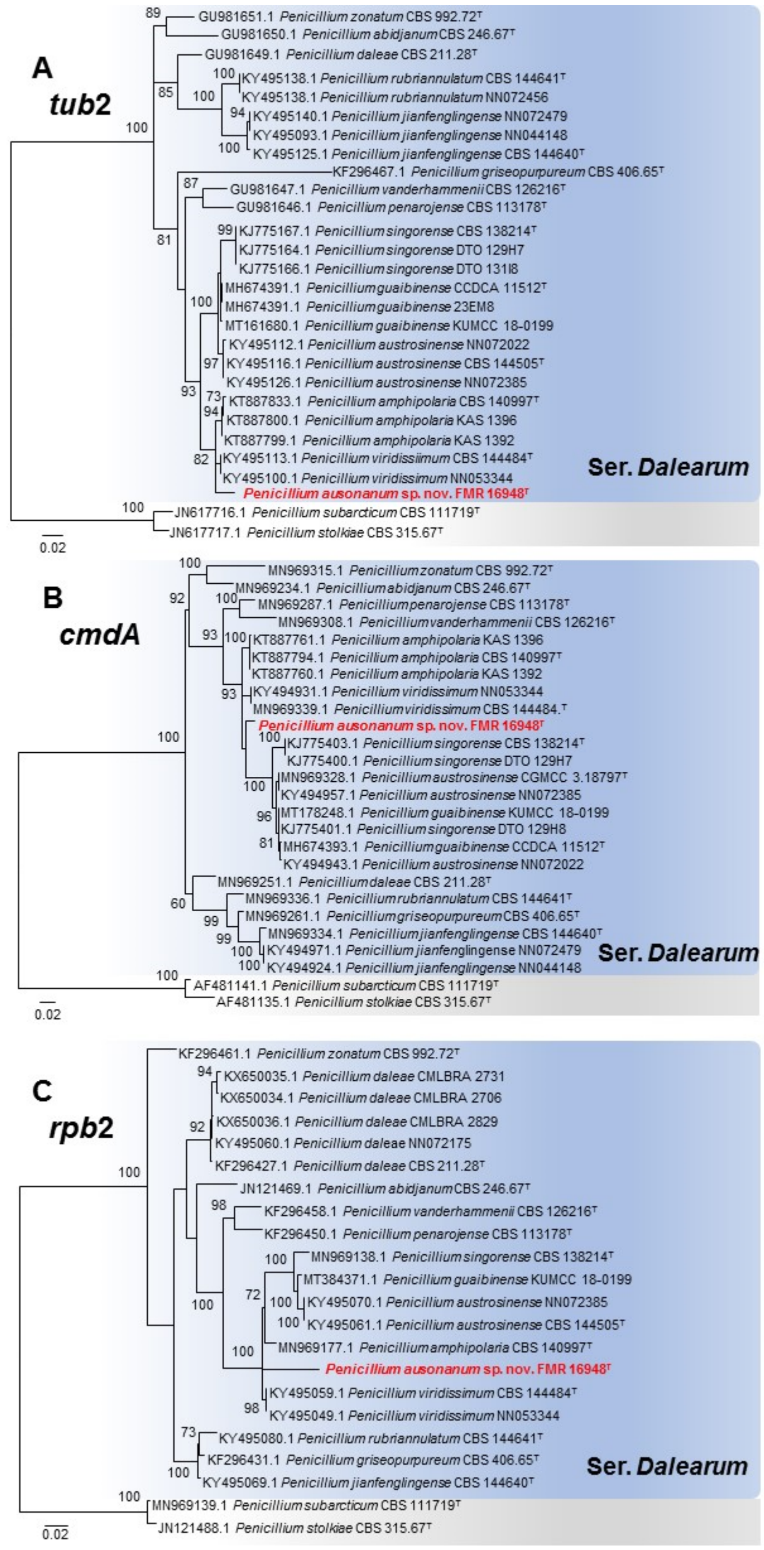

Figure S7: RAxML tree of selected strains of *Penicillium* in section *Lanata-Divaricata*, series *Dalearum*, inferred from (**A**) *tub*2, (**B**) *cmdA* and (**C**) *rpb*2. Determined by Mega software 6.0., K2+G was the best substitution model for *tub*2, K2+I for *cmdA* and TN93+G for *rpb*2.The aligned dataset was 526, 566 and 837 bp long for *tub*2, *cmdA* and *rpb*2, respectively, with 183 variable sites and 123 phylogenetically informative sites for *tub*2, 252 variable sites and 161 phylogenetically informative sites for *cmdA*, and 250 variable sites and 190 phylogenetically informative sites for *rpb*2. Branch lengths are proportional to phylogenetic distance. Bootstrap support values above 70% are indicated on the nodes. The trees are rooted to *P. subarcticum* CBS 111719 and *P. stolkiae* CBS 315.67 ^{T} = Ex-type strain.

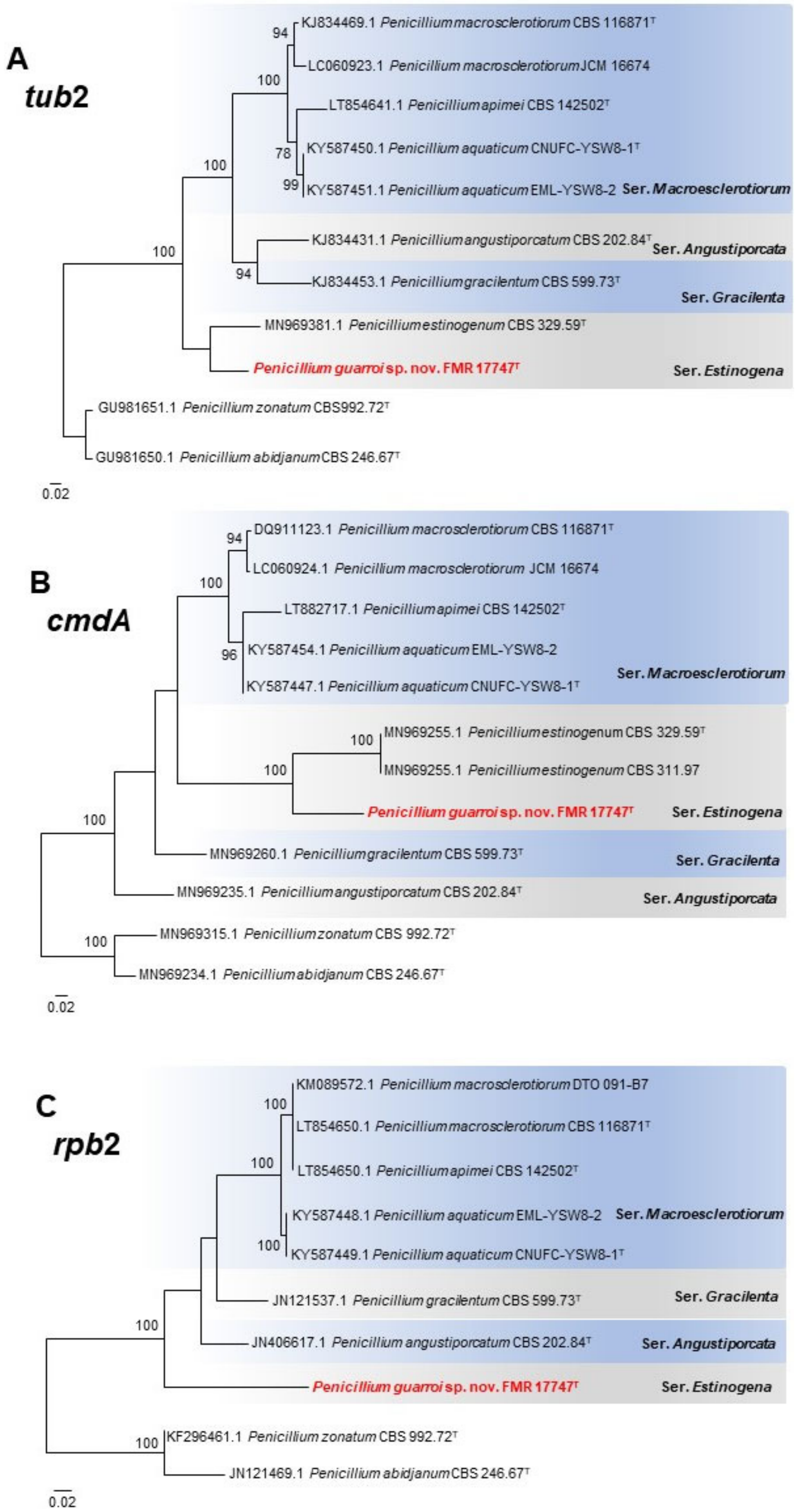

Figure S8: RAxML tree of selected strains of *Penicillium* in section *Gracilenta* inferred from (**A**) *tub*2, (**B**) *cmdA* and (**C**) *rpb*2. Determined by Mega software 6.0., K2+I was the best substitution model for *tub*2 and *cmdA*, and TN93+G for *rpb*2. The aligned dataset was 525, 643 and 952 bp long for *tub*2, *cmdA* and *rpb*2, respectively, with 201 variable sites and 128 phylogenetically informative sites for *tub*2, 277 variable sites and 202 phylogenetically informative sites for *cmdA*, and 288 variable sites and 184 phylogenetically informative sites for *rpb*2. Branch lengths are proportional to phylogenetic distance. Bootstrap support values above 70% are indicated on the nodes. The trees are rooted to *P. zonatum* CBS 999.72 and *P. abidjanum* CBS 246.67 ^{T} = Ex-type strain.

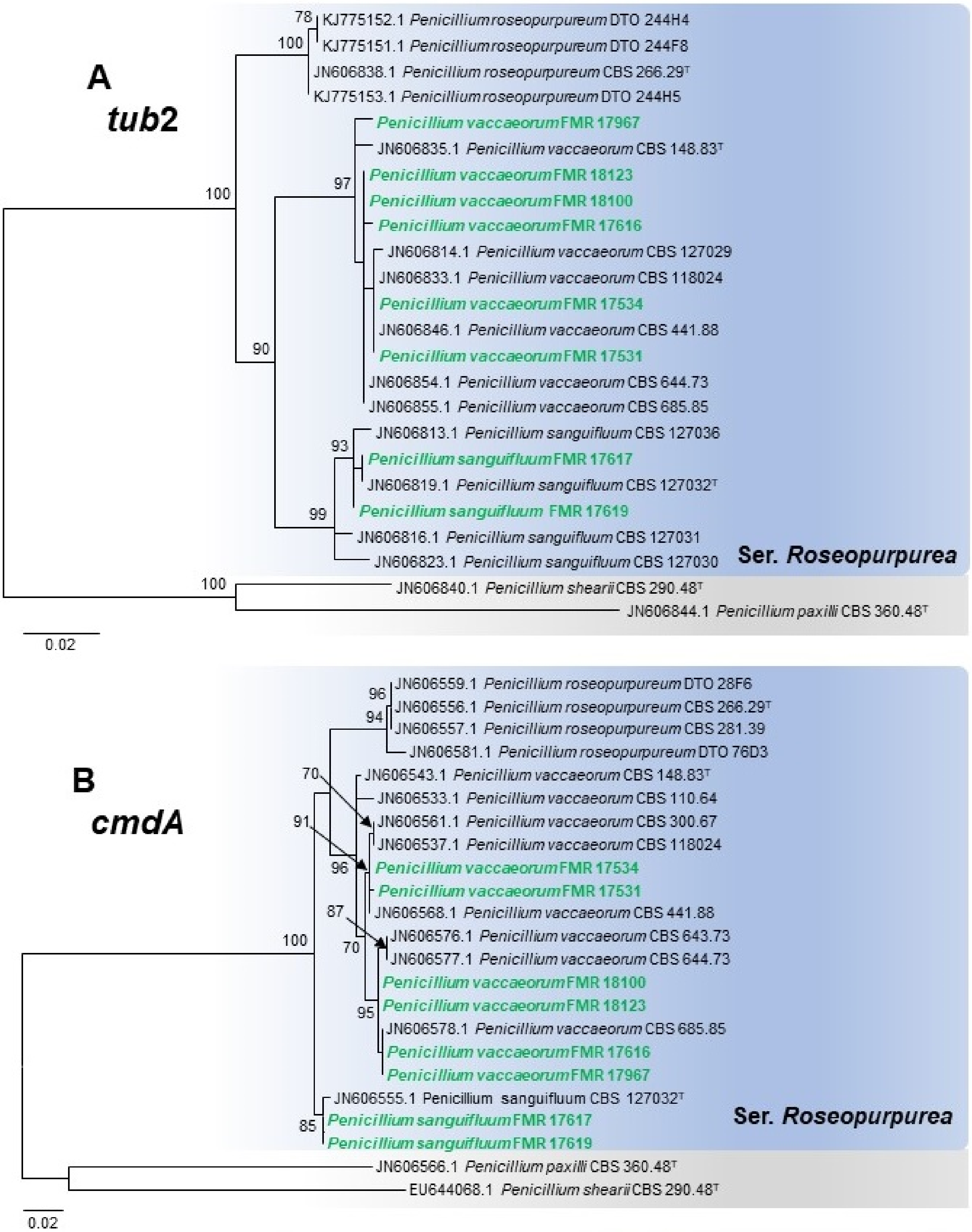

Figure S9: RAxML tree of selected strains of *Penicillium* in section *Citrina*, series *Roseopurpurea*, inferred from (**A**) *tub*2 and (**B**) *cmdA*. Determined by Mega software 6.0., K2+G was the best substitution model for both gene markers. The aligned dataset was 456 and 584 bp long for *tub*2 and *cmdA*, respectively, with 116 variable sites and 61 phylogenetically informative sites for *tub*2, and 173 variable sites and 68 phylogenetically informative sites for *cmdA*. Branch lengths are proportional to phylogenetic distance. Bootstrap support values above 70% are indicated on the nodes. The trees are rooted to *P. shearii* CBS 290.48 and *P. paxilli* CBS 360.48 ^{T} = Ex-type strain.
